# Microwave-Assisted Sol–Gel Preparation of the Nanostructured Magnetic System for Solid-Phase Synthesis

**DOI:** 10.3390/nano11123176

**Published:** 2021-11-24

**Authors:** Daniela Istrati, Alina Moroșan, Raluca Stan, Bogdan Ștefan Vasile, Gabriel Vasilievici, Ovidiu Oprea, Georgiana Dolete, Bogdan Purcăreanu, Dan Eduard Mihaiescu

**Affiliations:** 1 Department of Organic Chemistry “Costin Nenitescu”, Faculty of Applied Chemistry and Materials Science, University POLITEHNICA of Bucharest, 011061 Bucharest, Romania; daniela.istrati@upb.ro (D.I.); alina.morosan@upb.ro (A.M.); 2 Department of Science and Engineering of Oxide Materials and Nanomaterials, Faculty of Applied Chemistry and Materials Science, University POLITEHNICA of Bucharest, 011061 Bucharest, Romania; vasile_bogdan_stefan@yahoo.com (B.Ș.V.); dolete.georgiana@gmail.com (G.D.); 3 INCDCP-ICECHIM, 202 Spl. Independentei, 011061 Bucharest, Romania; gabi.vasilievici@gmail.com; 4 Department of Inorganic Chemistry, Physical Chemistry and Electrochemistry, Faculty of Applied Chemistry and Materials Science, University POLITEHNICA of Bucharest, 011061 Bucharest, Romania; ovidiu73@yahoo.com; 5 S.C. BIOTEHNOS S.A., Gorunului Rue, No. 3–5, 075100 Ilfov, Romania; bogdanpb89@gmail.com

**Keywords:** MW assisted synthesis, nanophase peptide synthesis (NphPS), core–shell magnetic nanoparticles, solid phase synthesis (SPhS)

## Abstract

This work describes a new synthesis method for core–shell magnetite nanoparticles with a secondary silica shell, functionalized with a linker system (Fe_3_O_4_-PABA-SiO_2_-linker) using a microwave-assisted heating technique. The functionalized solid nanomaterial was used for the nanophase synthesis of peptides (Fmoc route) as a solid support. The co-precipitation method was selected to obtain magnetite nanoparticles and sol–gel technique for silica coating using a microwave-assisted (MW) procedure. The magnetic properties of the nanoparticle core offer the advantage of a quick and easy alternative for the magnetic separation of the product from the reaction mixture, facilitating all the intermediary washing and separation operations. The intermediate and final materials were analyzed by advanced characterization methods. The effectiveness of the nanophase peptide synthesis using this nanostructured material as solid support was demonstrated for a short peptide sequence.

## 1. Introduction

Core–shell nanoparticles demonstrate significant advantages compared to classic materials due to their unique properties (high surface area, non-toxicity, low cost and biocompatibility) [1], related to important applications in the field of biomedicine, such as targeted drug delivery, magnetic resonance imaging (MRI), magnetic separation and many other fields of scientific or technological interests. The shell contributes to the improvement of their chemical and physical properties and ensures increased biocompatibility and biodegradability. Silica is the most widely used to functionalize the surface of nanoparticles [2], because it provides a steric barrier against core agglomeration preventing interactions between nanoparticles [3,4], good hydrophilicity (due to Si-OH), an increase in the stability of the dispersions over a wide pH range, and biocompatibility of the nanoparticles; it also allows the attachment of other functional groups for further application [5,6].

The silica shell can be synthesized by several methods, the most used being sol–gel and microemulsion [5]. The sol–gel method, first described by Ströber et al., offers the advantage of obtaining materials of high purity and homogeneity at low costs [5,7,8] and allows the control of shell thickness by adjusting the ratio of ammonium hydroxide concentration to tetraethyl orthosilicate (TEOS) [1]. The sol–gel method involves the use of an alkoxy precursor, the most widely used being TEOS as the main agent for the silica shell build-up, in the presence of a basic catalyst (ammonium hydroxide) [4]. The principle of the method consists of the TEOS hydrolysis rate control, by using magnetic nanoparticles as nucleation centers, so that after TEOS alkaline hydrolysis, the polycondensation reaction takes place on the magnetic nanoparticles surface. Ammonia is used as an alkaline agent, but it catalyzes the hydrolysis reaction of TEOS so rapidly that some silicic acid molecules cannot reach the surface of Fe_3_O_4_ particles before they agglomerate [5].

Several researchers have focused in recent years on nanophase peptide synthesis (NphPS) as a sustainable alternative to classic SPhS, mainly because of the significantly higher surface area and facilitated diffusional access of the used reagents at the reaction center and of significantly operational advantages [9,10,11].

In general, attachment of peptides on solid support is achieved via an ester or an amide bond, cleavage being performed using either acid or basic medium depending on the nature of the linker. In particular, when an HMBA linker is used, the peptide cleavage conditions in alkaline environment with sodium hydroxide [12], sodium methoxide in methanol, or other nucleophile-based cocktails determines a high purity of the resulted peptides; therefore, no further purification is needed before characterization and use. Additionally, acidic mixtures may be used for the esteric bond cleavage.

Microwave-assisted synthesis is a fast, low-cost, and a low-energy method for nanoparticle synthesis [13], with several advantages, such as reduced reaction time, high reproducibility, aggregation control and significantly improved diffusional access. Additionally, the use of MW ensures a homogeneous heating; the heat being evenly distributed in the solution, ensuring a narrow range size distribution and controlled physicochemical properties in the nanomaterial [14,15,16].

The present study is based on our previous work [9], related to nanophase peptide synthesis, focusing on the nanophase support synthesis optimization by an MW-assisted process, on more accessible linker systems and on the advantage of a higher purity of the final product by the TFA cleavage of the HMBA linker-peptide system. Additionally, an improved characterization of the final peptide by FT-ICR HR-MS analysis was performed, to prove the VIK sequence.

## 2. Materials and Methods

### 2.1. Chemicals and Reagents

All the reagents were used as received without any further purification. FeCl_3_ (ferric chloride), FeSO_4_·7H_2_O (ferrous sulfate heptahydrate), PABA (para-aminobenzoic acid), NaOH (sodium hydroxide), CTAB (cetyltrimethylammonium bromide), acetic acid, TEOS 98% (tetraethyl orthosilicate), ACN (acetonitrile), EtOH (ethanol), (3-aminopropyl) trimethoxysilane (APTMS), KCN (potassium cyanide), Nhy (ninhydrin), Py (pyridine), Fmoc-Lys(Boc)-OH ((2S)-2-(9H-fluoren-9-ylmethoxycarbonylamino)-6-[(2-methylpropan-2-yl)oxycarbonylamino]hexanoic acid), DMAP (4-dimethylaminopyridine), HMBA (4-(Hydroxymethyl)benzoic acid), DIC (*N*,*N*′- diisopropylcarbodiimide), diethyl ether, MeOH (methanol), PPy (piperidine) were purchased from Sigma Aldrich. HOBt (hydroxybenzotriazole), HBTU (2-(1H-benzotriazol-1-yl)-1,1,3,3-tetramethyluronium hexafluorophosphate) were purchased from Fluka (Buchs, Switzerland)). N-Fmoc-L-valine, Fmoc-L-isoleucine, DIPEA (N, N-diisopropylethylamine), DCM (dichloromethane) were purchased from Alfa Aesar (Heysham, Lancashire, UK.). DMF (N, N-dimethylformamide), TFA (trifluoroacetic acid), TIS (triisopropylsilane) were purchased from Merck Millipore (Darmstadt, Germany). Ultrapure water (18.2 MΩ) was used for all experiments.

Fourier transform infrared (FT-IR) spectra were recorded using an H-ATR with ZnSe crystal (Thermo Nicolet 6700 spectrometer, Thermo Fisher Scientific, Waltham, MA, USA). The measurements were carried out in the range of 4000–400 cm^−1^, using the resolution 8 cm^−1^ and co-adding 64 scans per each spectrum.

Dynamic Light Scattering (DLS) technique was used for particle size distribution using a Nano ZS Zetasizer (Malvern Instruments, Malvern, UK). Measurements were performed at a spreading angle of 90° and at a temperature of 25 °C; the reported values for average diameter and the polydispersity index are the average of three measurements.

Morphology of the magnetic nanoparticles was investigated using a QUANTA INSPECT F scanning microscope (SEM) ( Thermo Fisher—formerly FEI, Eindhoven, The Netherlands) equipped with EDX solid angle 0.13 (srad) at 1.2 nm resolution and Tecnai G2 F30 S-TWIN high-resolution transmission electron microscope (HR-TEM) equipped with energy dispersive spectroscopy (EDS) as well as a selected area electron diffraction detector (SAED) operated in transmission mode at 300 kV, with TEM point resolution 2 Å and 1 Å line resolution. For elemental analysis, X-ray dispersion energy at MnKα, 133 eV resolution was involved.

Thermal analysis (TD-DSC) was performed using a Netzsch STA 449C Jupiter (NETZSCH-Gerätebau GmbH, Selb, Germany) apparatus, by heating samples on open alumina crucibles, under a flow 50 mL min^−1^ dried air from 20 to 900 °C at 10 K·min^−1^ rate, using an empty alumina crucible as reference.

Brunauer–Emmett–Teller (BET) analysis. The nitrogen adsorption/desorption isotherms were recorded at 77 K in the relative pressure range p/po = 0.005–1.0, by using a NOVA 2200e Gas Sorption Analyzer (Quantachrome, Boynton Beach, FL, USA). Data processing was performed using NovaWin software. Prior to adsorption measurements, the samples were degassed up to 300 °C under vacuum. Standard Brunauer–Emmett–Teller (BET) equation was used to determine the specific surface area. The gas volume absorbed at a relative pressure p/po~1 allowed the estimation of the total pore volume. The Barrett–Joyner–Halenda (BJH) model was used to obtain the pore size distribution and mesopore volume from the desorption branch of the isotherm.

XR FTMS Hybrid System QqFTMS mass spectrometry with superconducting magnet-solariX XR 15T. The high-resolution mass spectrometry analysis was performed by using a Fourier Transform–ion cyclotron resonance (FT-ICR) spectrometer, SolariX XR 15T (Bruker Daltonics, Bremen, Germany). The sample was introduced by direct infusion, positive ESI ionization, with a sample flow rate of 200 µL/h, with a nebulization gas pressure (N_2_) of 3 Barr at 200 °C and a flow rate of 6 L/min. The spectra were recorded by MS-MS (using monoisotopic peak isolation), over a mass range between 46 and 1500 amu at a source voltage of 5500 V.

Microwave thermal treatment (Stuttgart, Germany) was performed in a MW4717, tated microwave power output 600 W, microwave frequency 2450 MHz.

A vibrating sample magnetometer VSM-7400 Lake Shore (Westerville, OH, USA) operating up to 12.000 Oe was used for recording of the magnetic properties of the obtained nanoparticles.

Magnetic separation: Separation of the magnetic nanoparticles was performed using a NdFeB magnet.

### 2.2. Preparation

#### 2.2.1. Synthesis of Nanostructured System

**Scheme 1:**
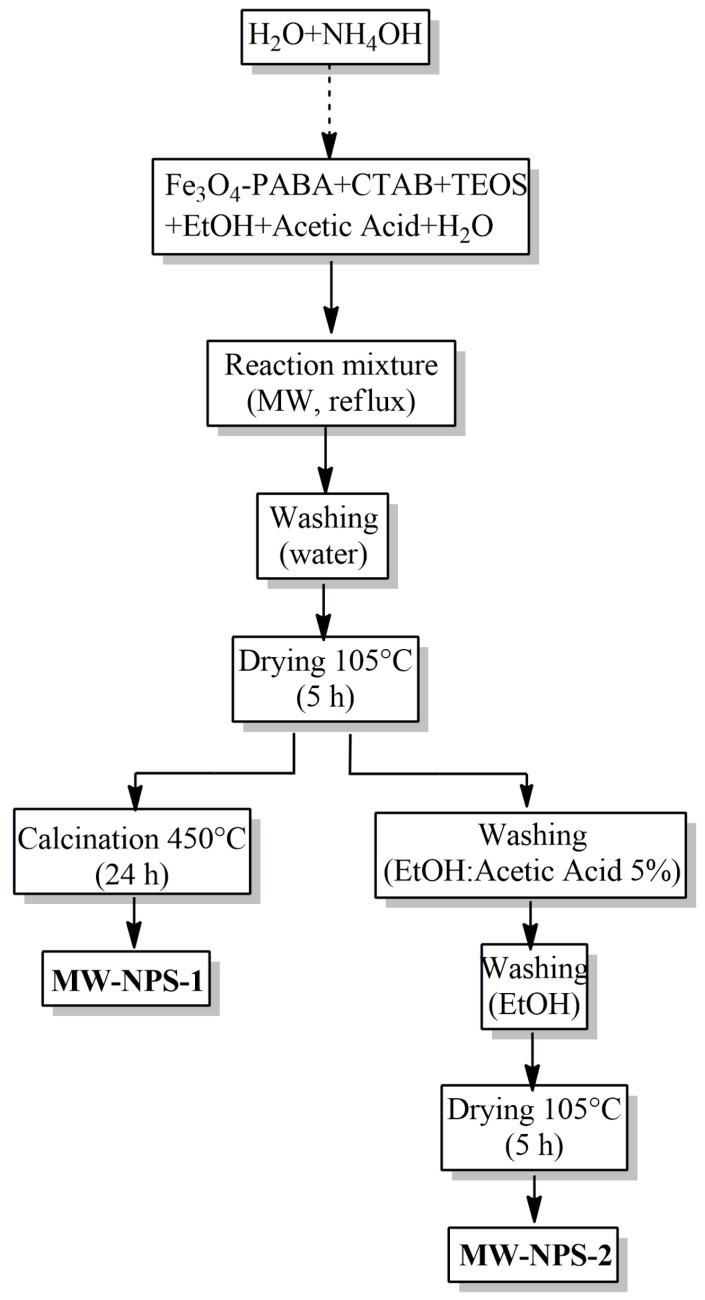
Flow chart for obtaining **MW-NPS-1** and **MW-NPS-2**.

**Scheme 2:**
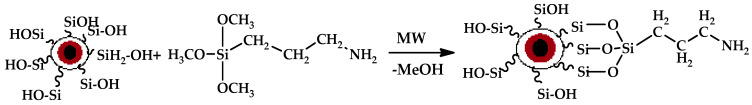
Synthesis of MW-NPS-APS.

**Scheme 3:**
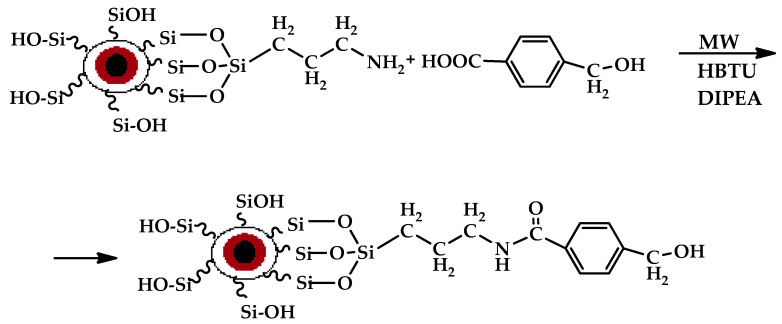
Synthesis of the MW-NPS-APS-HMBA.

The Fe_3_O_4_–PABA was prepared using the co-precipitation method. The second step in the synthetic route was based on the microwave-assisted reaction to obtain the Fe_3_O_4_–PABA-SiO_2_ (MW-NPS, Scheme 1) nanoparticles with relatively uniform particle size, using a modified microwave-assisted sol–gel homogeneous precipitation method with NH_4_OH as the alkaline agent, CTAB as the surfactant, and TEOS as the silica source. Microwave-assisted synthesis ensures an efficient diffusional reagent transfer, as well as an efficient silica precursor polycondensation reaction. Both the third and the final synthetic step were also accomplished by microwave heating, respectively with (3-aminopropyl) trimethoxysilane and 4-(hydroxymethyl) benzoic acid, ensuring a higher diffusion rate in the material and an easier transfer of reactants at the reaction center (Scheme 2 and Scheme 3).

a. Synthesis of magnetic core–shell nanoparticles (Fe_3_O_4_-PABA)

The synthesis of magnetic core nanoparticles was accomplished by the co-precipitation method, as reported earlier [17], and characterized by FT-IR, DLS, SEM and TEM.

b. Core-shell Fe_3_O_4_-PABA-SiO_2_ nanoparticle synthesis

The magnetic SiO_2_ shell nanoparticles with secondary silica shell were synthetized using a modified sol–gel method. A 150 mL volume of the resulting **Fe_3_O_4_-PABA** dispersion was mixed with 150 mL ethanol, 3 g CTAB, 3 mL acetic acid, 0.9 mL TEOS and 300 mL H_2_O were heated using microwave irradiation (MW). A solution consisting of 15 mL NH_4_OH and 10 mL H_2_O was added dropwise for 20 min. After 10 min reaction time, the product **Fe_3_O_4_-PABA-SiO_2_** (**MW-NPS**) was magnetically separated, washed several times with ultra-pure water, and dried at 105 °C. In the last stage, 150 mg **MW-NPS** was heated to 450 °C for 24 h and named **MW-NPS-1**; and another 150 mg **MW-NPS** was washed with ethanol: acetic acid 5% (5 × 40 mL) and further with ethanol (1 × 40 mL), and named **MW-NPS-2**.

c. Synthesis of Fe_3_O_4_-PABA-SiO_2_-APS (MW-NPS-APS)

With respect to surface functionalization, **MW-NPS** was treated with (3-aminopropyl) trimethoxysilane (APTMS). Briefly, 120 mg **MW-NPS**, 30 mL CH_2_Cl_2_ and 5 mL APTMS were heated using microwave irradiation (MW) for 30 min, washed several times with ultra-pure water, and dried at 105 °C.

d. Synthesis of Fe_3_O_4_-PABA-SiO_2_-APS-HMBA (MW-NPS-APS-HMBA)

The final nanostructured system, **MW-NPS-APS-HMBA**, was obtained by dissolving 183 mg HMBA in 4 mL DMF, 417 mg HBTU and 348 μL DIEA. This mixture was added to 80 mg **MW-NPS-APS** and heated using microwave irradiation (MW) for 15 min. The resulting functionalized nanoparticles (**MW-NPS-APS-HMBA**) were washed with DMF (4 × 4 mL, 10 min shaking), and dried at 105 °C. For all intermediate stages, the magnetic core nanoparticles were separated from the reaction mixture by an external magnetic field.

#### 2.2.2. The Peptide Synthesis

The peptide synthesis was performed according to a previously described protocol [9], by a nanophase step by step AA (amino acids) chain build-up, followed by the final TFA cleavage. The crude reaction product (**Val-Ile-Lys**) was analyzed using direct sample introduction (syringe infusion) by HR-MS (FT-ICR).

## 3. Results and Discussion

### 3.1. Nanostructured System

DLS, TEM and SEM advanced characterization methods were used in order to prove the **Fe_3_O_4_-PABA** nanoparticles’ diameter, crystalline structure, and the related surface morphology.

The specific structure of the **Fe_3_O_4_-PABA** nanoparticles with NH_3_^+^ groups in the outer shell (from p-aminobenzoic acid primary shell) determined an average diameter of 47 nm, a good size homogeneity (polydispersity index 0.114), and a significant stability of the colloidal system (zeta potential +54 mV) as determined by DLS measurement (Figure S1 in the Supplementary Materials).

**Figure 1:**
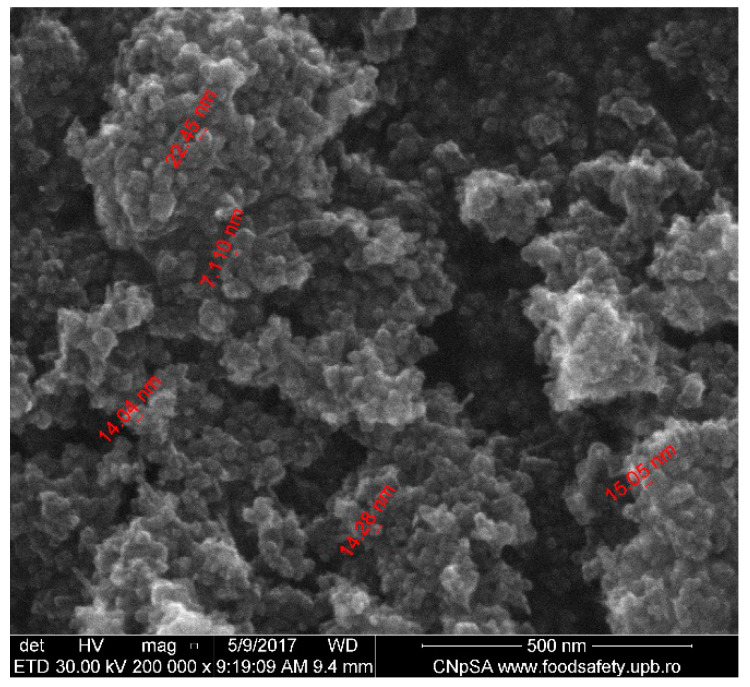
The SEM image of Fe_3_O_4_-PABA.

The SEM image of **Fe_3_O_4_-PABA** nanoparticle (Figure 1) indicates a granular morphology with good homogeneity and a uniform grain size and shape distribution on the surface.

**Figure 2:**
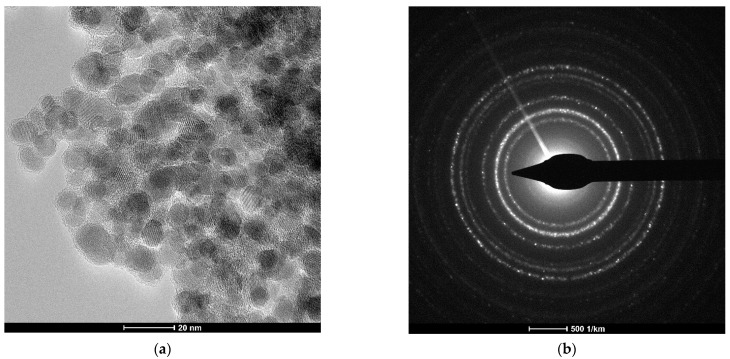
(**a**) TEM and (**b**) SAED images of magnetite nanoparticles with PABA shell.

The TEM image (Figure 2a) confirms the presence of nanoparticles, and that the size of the ferrite core is below 12 nm. The Selected Area Electron Diffraction (SAED) image (Figure 2b) proved the presence of the Fe_3_O_4_ crystalline structure.

**Figure 3:**
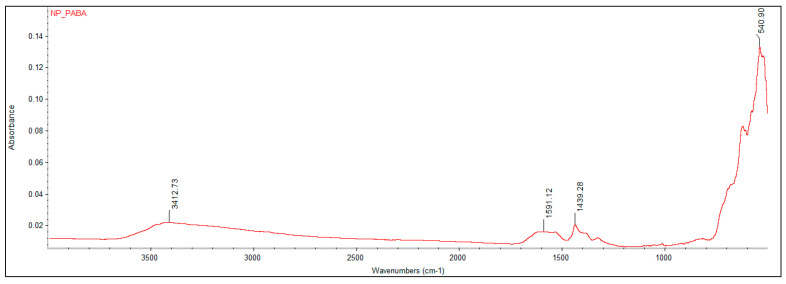
The FT-IR spectrum of **Fe_3_O_4_-PABA**.

The presence of the organic layer coating the magnetic nanoparticles **Fe_3_O_4_-PABA** is proved both by the elemental composition (Fe, O, C and N) provided by Energy dispersive X-ray spectroscopy (EDX) (Figure S2 in the Supplementary Materials) and by the characteristic absorption bands in the FT-IR spectrum (Figure 3). The most representative bands are the stretching of the free –NH_3_^+^ group at 3412 cm^−1^, asymmetric stretching vibrations of carboxylate groups at 1591 cm^−1^, and the O-H bending band from the carboxylic group at 1439 cm^−1^. The characteristic band assigned to the Fe-O stretching can be observed at 540 cm^−1^.

BET analysis (Figures S3 and S4 in the Supplementary Materials) provided the specific surface area of the **Fe_3_O_4_-PABA** nanoparticles. The specific surface of the obtained material was 124 m^2^ g^−1^, with pore size and volume dBJH = 12.019 nm and Vp = 0.295 cm^3^ g^−1^. To obtain an efficient surface functionalization of the final composite nanoparticles, a further silica shell was obtained (**MW-NPS**). The silanolic rich surface provides an easy and efficient subsequent functionalization [18]. As determined by magnetic measurements, the final material retains its magnetic properties, which allows easy and fast extraction of intermediate and final products from the reaction mixture, greatly facilitating all intermediate operations.

A modified sol–gel method was used for the synthesis of the **MW-NPS** nanoparticles in microwave field. In the adopted procedure, TEOS was used as a source of silica for the formation of the secondary shell, NH_4_OH as catalyst, and CTAB as surfactant, the reaction mixture being heated to reflux by microwave irradiation. The disadvantage of using CTAB as a template is related to the removal process by calcination or washing. The advantage of calcination is the complete template elimination; however, the processing operations involve a long time, high costs, and a specific surface area mitigation at higher temperatures. By washing, the template is partially removed, yielding an intact silica shell and low nanoparticle aggregation.

The secondary silica shell was obtained according to the procedure shown in Scheme 1. For the **MW-NPS-1** sample, the template was removed by thermal treatment at a temperature of 450 °C for 24 h; for the **MW-NPS-2** sample, the template was partially removed by subsequent washing with EtOH solution 5% acetic acid and EtOH, respectively.

The structure and morphology of **MW-NPS-1** and **MW-NPS-2** nanoparticles was investigated using advanced characterization techniques: TEM, SAED, FT-IR, EDX.

**Figure 4:**
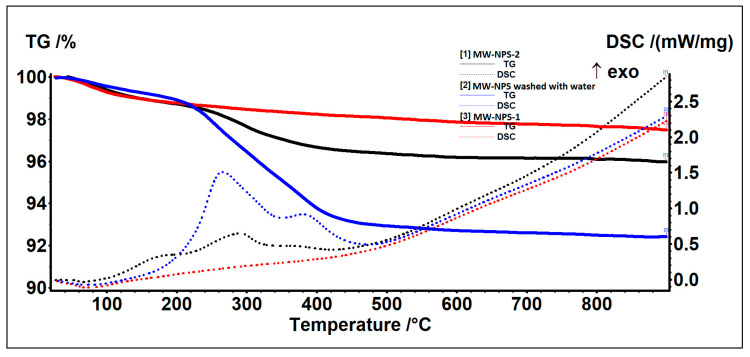
The TGA analysis of **MW-NPS** after the water washing, **MW-NPS-1** and **MW-NPS-2**.

The efficiency of the template removal process was highlighted by TGA analysis (Figure 4) of the core–shell nanoparticles after washing with ultrapure water, calcination, respectively, washing with organic solvent (Figures S5–S7 in the Supplementary Materials). According to the literature data related to TGA analysis, the template used in synthesis, CTAB is stable up to 210 °C, after which it undergoes a sudden decomposition process up to 380 °C [19]. The same effect also occurs through decomposition of the PABA, where this process begins after 150 °C and is almost completed by around 230 °C [20].

TGA analysis (Table 1) indicated that the organic solvent washing step removes the CTAB template more efficiently than washing with water, which is visible due to the higher mass loss (in the case of washing with water) in the range of 190–500 °C, when a large amount of substance is oxidized. The **MW-NPS-1** sample shows the lowest mass loss, with the volatile or easily oxidizable substances already being removed by the calcination process.

No color change or changes of magnetic properties were observed for any of the resulting residues after TGA analysis of MW-NPS, proving the presence of the silica secondary shell, and also sustained by the absence of specific physical transformation of maghemite–hematite in the range of 500–700 °C [21].

For porous silica materials, the final C level increases by the increase of the organic matrix concentration and the mitigation of oxygen diffusional access and a carbon deposition on the silica material can occur. In the calcination process of MW-NPS-1, the PABA shell of magnetite nanoparticles was removed, and the C level was lowered compared to MW-NPS-2. A thermal decomposition and a transition to carbon for the inner sphere from PABA is to be expected, which is related to the hindered oxygen diffusional access, together with no carbon deposition on the outer sphere due to the external surface access to oxygen. We estimate that this process will not affect the structural stability of the nanocomposite support, or the silanolic activity of the external surface, involved in the functionalization process. As the TGA mass loss for MW-NPS-1 is really low for the 190–500 °C range, we estimate that the PABA decomposition was complete by calcination, and the 500–900 °C mass loss can be assigned to carbon loss (air-purge experiment).

The specific surface areas of **MW-NPS-1** and **MW-NPS-2** were obtained by BET analysis (Figures S8–S11 in the Supplementary Materials). The specific surface area for the **MW-NPS-1** sample is 127 m^2^ g^−1^, pore size dBJH = 33.223 nm and pore volume Vp = 0.690 cm^3^ g^−1^; and for the **MW-NPS-2** the specific surface area is 122 m^2^ g^−1^ with pore size dBJH = 19.497 nm and pore volume Vp = 0.595 cm^3^ g^−1^.

From Table 2 it can be seen that the template removal procedure for MW synthesized samples does not significantly affect the specific surface area or pore volume. A significant difference is related to the pore diameter, which is larger for the calcined sample due to the incomplete removal of the organic template for the washed samples, in correlation with the thermogravimetric analysis results (presented above).

The presence of a silica shell both for the **MW-NPS-1** and **MW-NPS-2** nanoparticles was confirmed by characteristic absorption bands in FT-IR spectra (Figures S12 and S13 in the Supplementary Materials), as follows: characteristic absorption for asymmetric Si-O-Si stretching vibrations at 1049 cm^−1^ and 1064 cm^−1^, respectively. The characteristic band of magnetite can be observed at 551 cm^−1^ for MW-NPS-1 and 553 cm^−1^ for MW-NPS-2, respectively, corresponding to the Fe-O bond vibration.

**Figure 5:**
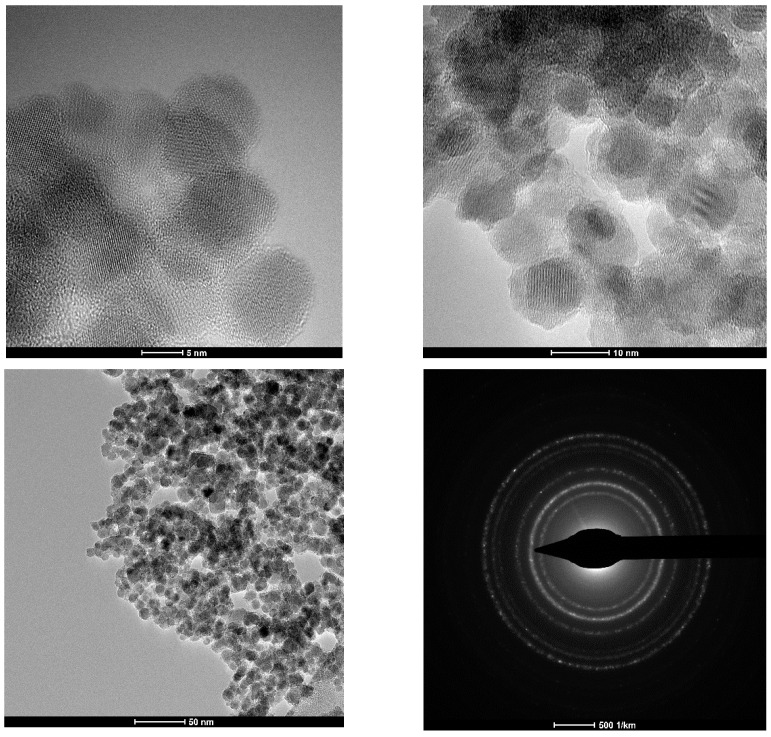
TEM and SAED images for **MW-NPS-1** nanoparticles.

The morphology of the **MW-NPS-1** nanoparticles was determined by TEM (Figure 5). The presence of the silica shell, with a thickness of approximately 2 nm, was proved by analyzing the obtained micrographs. The SAED image confirms the crystal structure of Fe_3_O_4_.

**Figure 6:**
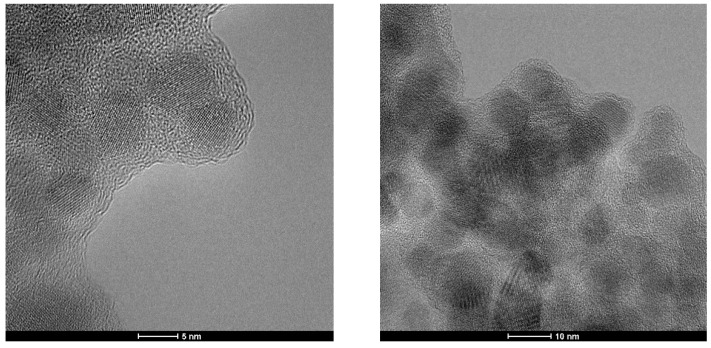

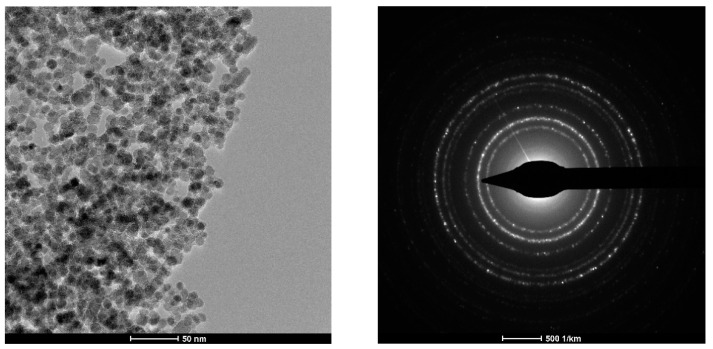
TEM and SAED images for **MW-NPS-2**.

The morphology of the **MW-NPS-2** nanoparticles was determined by TEM (Figure 6). The obtained images confirm the presence of the silica shell with a thickness of approximately 2 nm. The crystal structure of Fe_3_O_4_ is confirmed by the SAED image.

The elemental composition of the **MW-NPS-1** and **MW-NPS-2** nanoparticles performed using energy dispersive X-ray spectroscopy (EDX) (Figures S14 and S15 in the Supplementary Materials), confirms the secondary silica shell of the obtained nanoparticles.

The magnetic properties of **MW-NPS-2** nanoparticles (Figure S16 in the Supplementary Materials) show a value of saturation magnetization Ms = 57.121 emu g^−1^. The obtained nanoparticles were efficiently separated using a neodymium magnet.

Related to the magnetite core of the nanostructured support, we estimate that mild or strong alkaline environment will not affect the core but will affect the silica secondary shell, despite the surface functionalization. Additionally, strong acidic environment will be able to affect the magnetic core of the calcinated product MW-NPS-1, as the PABA initial shell proves to be an effective protection of the core for the MW-NPS-2.

Functionalization of silica secondary shell of **MW-NPS-1** and **MW-NPS-2** magnetic nanoparticles with free reactive -NH_2_ groups required for HMBA attachment was performed with 3-aminopropyltrimethoxysilane (APTMS) [16].

The **MW-NPS-APS** was obtained using a labile and commonly used linker in solid support peptide synthesis namely 4-(hydroxymethyl)benzoic acid (HMBA).

The functionalization with APTMS of **MW-NPS-1** and **MW-NPS-2** nanoparticles was confirmed by the FT-IR spectra (Figures S17 and S18 in the Supplementary Materials) by the presence of absorption bands characteristic of the free -NH_2_ groups at 3361 and 3202 cm^−1^, and 3429 and 3229 cm^−1^, respectively, and of the band corresponding to the stretching vibration CH at 2931 cm^−1^ and 2929 cm^−1^, respectively. Additionally, the characteristic bands of asymmetric stretching vibrations of Si-O-Si at 1032 cm^−1^ and 1040 cm^−1^, respectively, can be observed, and the characteristic band of magnetite can be observed at 549 cm^−1^ and 541 cm^−1^, respectively, corresponding to the Fe-O.

The magnetic properties of **MW-NPS-2-APS** (Figure S19 in the Supplementary Materials) nanoparticles indicate a value of saturation magnetization of Ms = 50.883 emu g^−1^. The obtained material was also efficiently separated using a neodymium magnet.

**Figure 7:**
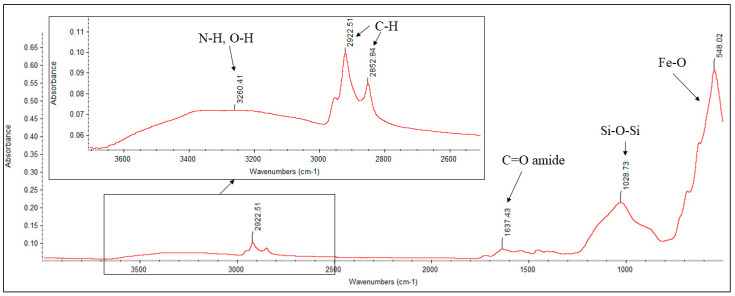
FT-IR spectrum for MW-NPS-1-APS-HMBA.

**Figure 8:**
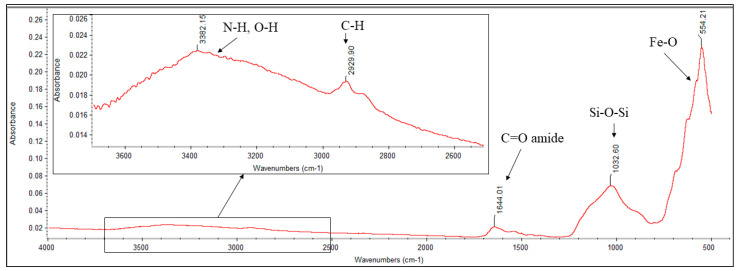
FT-IR spectrum for MW-NPS-2-APS-HMBA.

Obtaining of the **MW-NPS-1-APS-HMBA** and **MW-NPS-2-APS-HMBA** nanostructured systems was confirmed by FT-IR spectra (Figure 7 and Figure 8) by the presence of characteristic bands of the N-H and O-H stretching vibrations at 3260 cm^−1^ and 3382 cm^−1^, respectively, and the band corresponding to the C-H stretching vibration at 2922 and 2852 cm^−1^, and 2929 cm^−1^, respectively. Additionally, the band corresponding to the C=O stretching vibration of the secondary amide at 1637 cm^−1^ and 1644 cm^−1^, respectively, and the characteristic bands of the asymmetric stretching vibration of Si-O-Si at 1028 cm^−1^ and 1032 cm^−1^, respectively, can be found. The characteristic band of magnetite corresponding to the Fe-O bond can be observed at 548 cm^−1^ and 554 cm^−1^, respectively, for the two materials.

### 3.2. Peptide Synthesis

The efficiency of the newly synthesized nanostructured system **MW-NPS-2-APS-HMBA** was highlighted by using it as a solid support in nanophase peptide synthesis (nanostructured support synthesis—NSS, Table 3). The use of magnetically nanostructured solid support overcomes both the problem of solubility, giving the advantage of the access of the reactants to the reaction center and a rapid separation of intermediate and final products from the reaction medium in the magnetic field.

The peptide sequence **Val-Ile-Lys** was synthesized according to a protocol described earlier [9] using as solid support the nanostructured system **MW-NPS-2**.

The synthesized peptide sequence **Val-Ile-Lys** can be found in the decapeptide Val-Ile-Lys-Lys-Ser-Thr-Ala-Leu-Leu-Gly, which belongs to the class of water-soluble oligopeptides with pharmacological anti-inflammatory properties [22].

**Figure 9:**
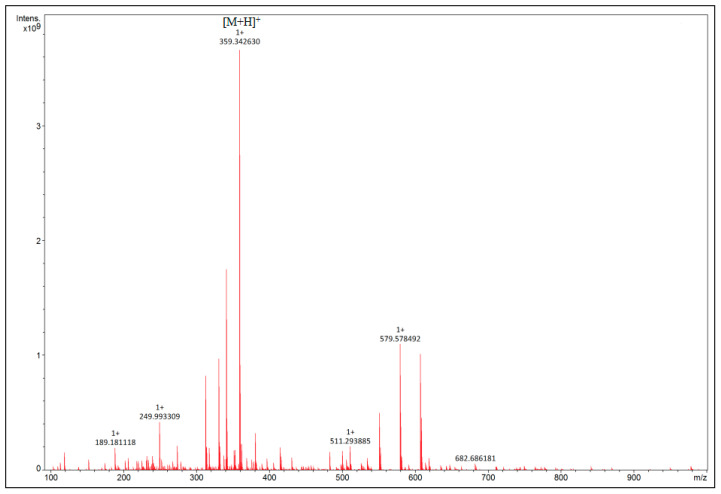
HR-MS analysis of **Val-Ile-Lys** peptide after cleavage from the **MW-NPS-2-APS-HMBA** nanostructured system (first screening before the MS-MS experiment).

The structure of the obtained tripeptide **Val-Ile-Lys (VIK)** was confirmed by the mass spectrum (Figure 9) obtained by HR-MS using the ESI ionization technique. The protonated molecular ion [M+H]^+^ at *m/z* 359.34 can be observed according to the values calculated using PROTEOMICS TOOLKIT. Additionally, the peaks at *m/z* 381.32 corresponding to [M+Na]^+^ and *m/z* 397.30 corresponding to [M+K]^+^ could be observed (Figure S20 in the Supplementary Materials).

The isotopic peaks of type ([M+H]^+^ + 1), ([M+H]^+^ + 2), ([M+H]^+^ + 3) from the protonated molecular ion [M+H]^+^ were also identified by the first screening before the MS-MS experiment (Figure S21 in the Supplementary Materials).

**Figure 10:**
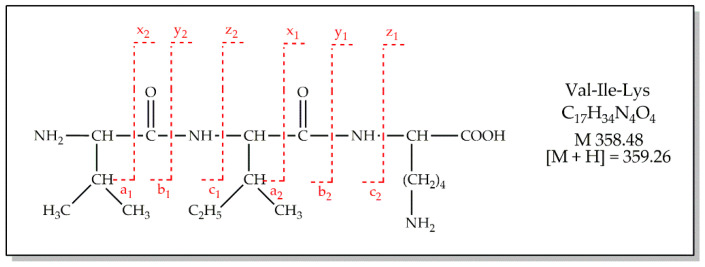
Theoretical fragmentation of the **Val-Ile-Lys** peptide.

The structure was also confirmed by examining the characteristic fragmentation of the tripeptide according to the general scheme presented in Figure 10. The theoretical and identified experimental fragments are presented in Table 4 and Table 5.

In the MS-MS spectra (Figures S22–S26 in the Supplementary Materials) the following types of fragments were identified corresponding to the **VIK** peptide: **c_1_**, **y_1_**_,_ **z_1_** with *m/z* 117.43, 130.16, 147.99, respectively; **x_1_** and **a_2_** with *m/z* 173.00, 185.92, respectively; **b_2_**, **c_2_** and **z_2_** with *m/z* 213.90, 230.99, 243.80, respectively; **x_2_** and **y_2_** with *m/z* 286.82 and 260.88, respectively; and [M+H-H_2_O]^+^, [M+H-CO-H_2_O]^+^, [M+H-NH_3_]^+^, [M-H_2_O+Na]^+^, [M+H-2H_2_O]^+^ with *m/z* 341.26, 313.22, 342.26, 363.30, 323.24, respectively, proving the successful synthesis of the desired compound.

**Scheme 4:**
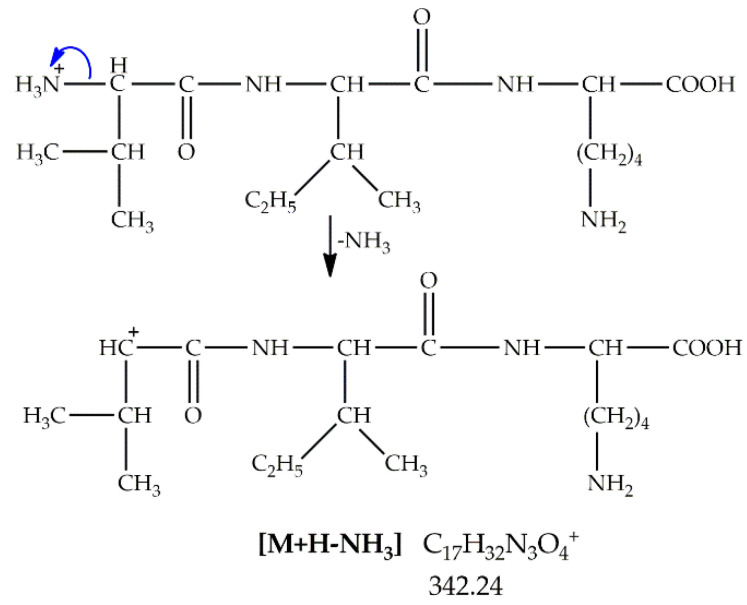
Possible mechanism of fragment formation [M+H-NH_3_]^+^.

**Scheme 5:**
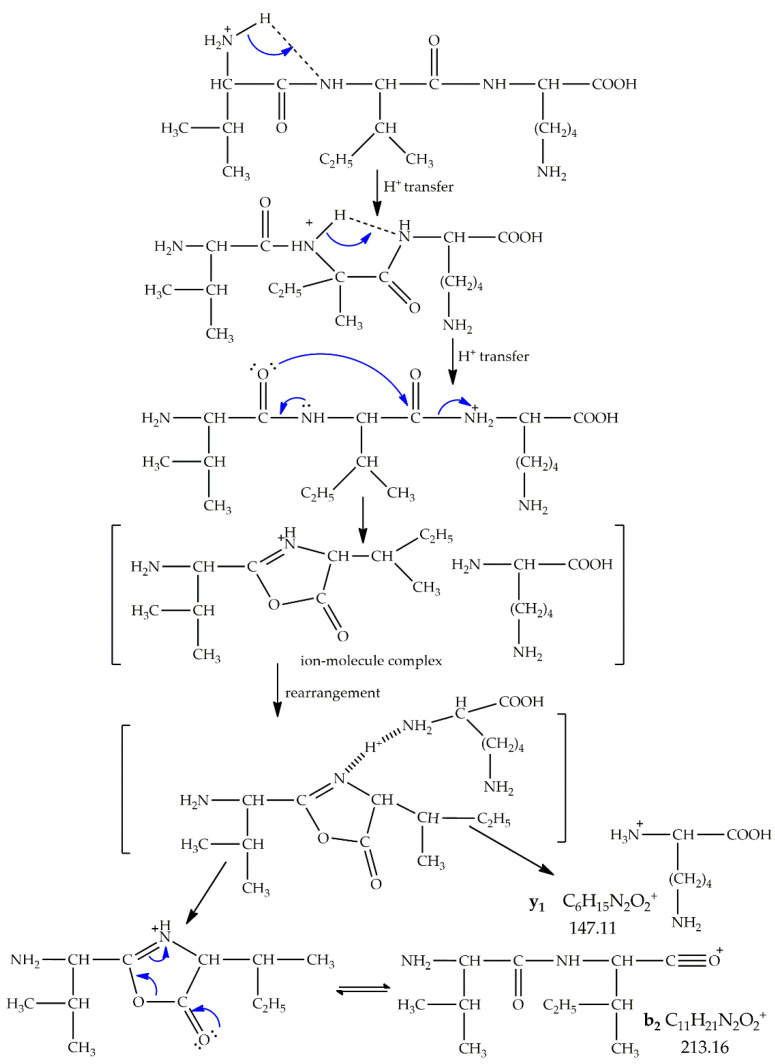
Fragmentation mechanism leading to **b_2_** and **y_1_** fragments.

**Scheme 6:**
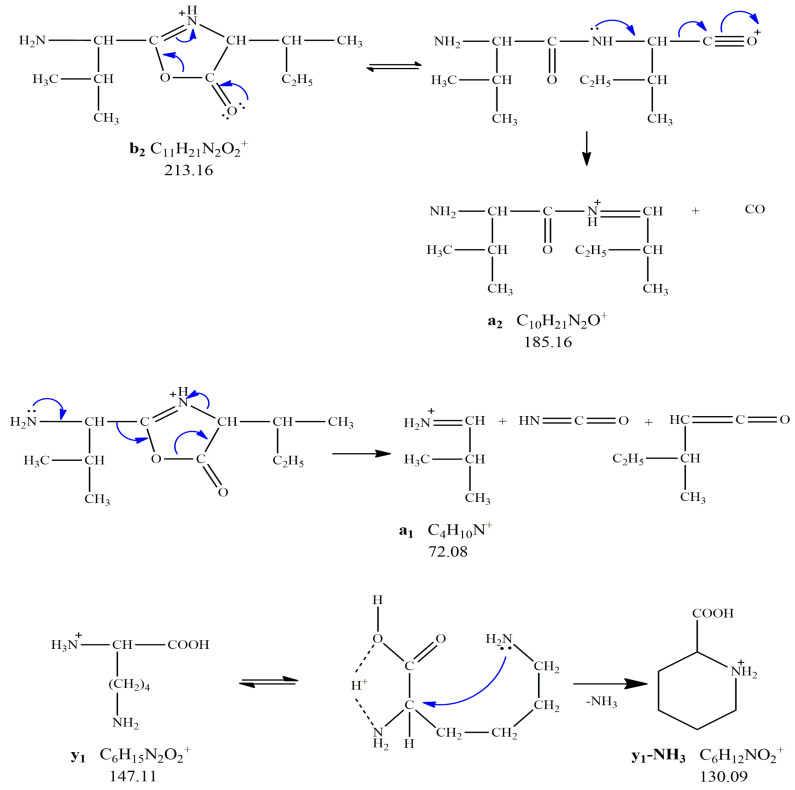
Fragmentation mechanism leading to **a_1_**, **a_2_** and **y_1_-NH_3_** fragments.

**Scheme 7:**
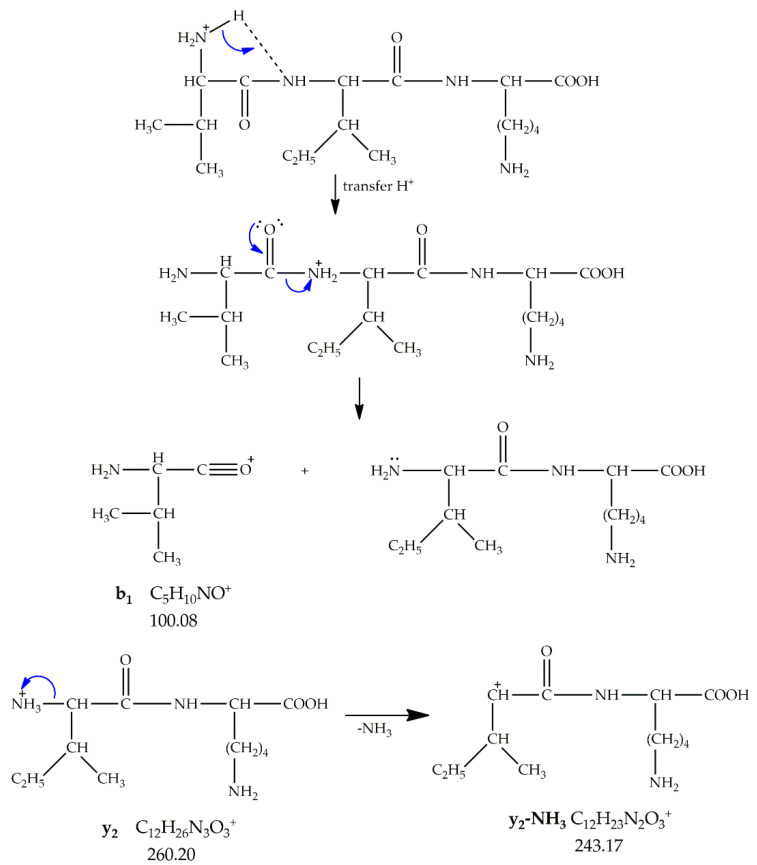
Fragmentation mechanism leading to **b_1_** and **y_2_-NH_3_** fragments.

For the identified fragments type [M+H-NH_3_]^+^, **b_1_**, **b_2_**, **y_1_**, **a_1_**, **a_2_**, **y_1_-NH_3_**, **y_2_-NH_3_** possible formation mechanisms are proposed in Scheme 4, Scheme 5, Scheme 6 and Scheme 7, adapted from the literature [23,24]:

## 4. Conclusions

A new magnetic nanostructured support system based on core–shell Fe_3_O_4_-PABA nanoparticles with a secondary silica shell, functionalized with a new linker system with free –OH group, obtained via APTMS and HMBA, was designed and synthesized by a MW-assisted route. The ability to provide efficient peptide nanophase synthesis of this new nanostructured magnetic support system was tested in a successful coupling with three Fmoc protected amino acids. The specific MS-MS fragmentation of the monoisotopic peak from the VIK peptide, by HR-MS FT-ICR, proves the proposed AA sequence.

The proposed MW-assisted method provides an efficient route to nanophase support synthesis, and an effective tool in nanophase peptide synthesis. These new magnetic core nanomaterials are potential candidates for the development of nanophase supports as alternative materials to the classic solid phase synthesis, with significant operational and technological advantages.

There is a significant potential related to the extension of nanophase peptide synthesis to different HMBA cleavage approaches or other linker pathways, and also to nanophase synthesis approaches in organic chemistry.

## Figures and Tables

**Table 1:** Experimental data obtained from TGA analysis.

Samples		RT		Residue	Endo	Exo I	Exo II	Exo III
	RT-190	190–500	500–900					
**MW-NPS washed with water**	1.01%	6.06%	0.51%	92.41%	74.2 °C	-	265.8 °C	379.5 °C
**MW-NPS-1**	1.23%	0.73%	0.56%	97.48%	73.2 °C	-	-	-
**MW-NPS-2**	1.25%	2.39%	0.39%	95.97%	65.2 °C	170.0 °C	290.4 °C	373.6 °C

**Table 2:** BET analysis for Fe_3_O_4_-PABA, MW-NPS-1 and MW-NPS-2 samples.

Samples		BET Analysis	
	SBET	dBJH	Vp
**Fe_3_O_4_-PABA**	124 m^2^ g^−1^	12.019 nm	0.295 cm^3^ g^−1^
**MW-NPS-1**	127 m^2^ g^−1^	33.223 nm	0.690 cm^3^ g^−1^
**MW-NPS-2**	122 m^2^ g^−1^	19.497 nm	0.595 cm^3^ g^−1^

**Table 3:** The NSS strategy.

Magnetic Nanostructured Support	Peptide Sequence	Chemical Formula	MW (g mol^−1^)
**MW-NPS-2-APS-HMBA**	Val-Ile-Lys (VIK)	C_17_H_34_N_4_O_4_	358.46

**Table 4:** Types of fragments identified in the MS spectra corresponding to the **VIK** peptide.

Fragment Type	Chemical Formula	*m/z* Theoretical	*m/z* Experimental
**a_1_**	C_4_H_10_N^+^	72.08	-
**a_2_**	C_10_H_21_N_2_O^+^	185.16	185.92
**b_1_**	C_5_H_10_NO^+^	100.08	-
**b_2_**	C_11_H_21_N_2_O_2_^+^	213.16	213.90
**c_1_**	C_5_H_13_N_2_O^+^	117.10	117.43
**c_2_**	C_11_H_24_N_3_O_2_^+^	230.19	230.99
**x_1_**	C_7_H_13_N_2_O_3_^+^	173.09	173.00
**x_2_**	C_13_H_24_N_3_O_4_^+^	286.18	286.82
**y_1_**	C_6_H_15_N_2_O_2_^+^	147.11	147.99
**y_2_**	C_12_H_26_N_3_O_3_^+^	260.20	260.88
**z_1_**	C_6_H_12_NO_2_^+^	130.09	130.16
**z_2_**	C_12_H_23_N_2_O_3_^+^	243.17	243.80

**Table 5:** Additional types of fragments identified for **Val-Ile-Lys** peptide.

Fragment Type	Chemical Formula	*m/z* Theoretical	*m/z* Experimental
**[M+H-H_2_O]^+^**	C_17_H_33_N_4_O_3_^+^	341.25	341.26
**[M+H-CO-H_2_O]^+^**	C_16_H_33_N_4_O_2_^+^	313.26	313.22
**[M+H-NH_3_]^+^**	C_17_H_32_N_3_O_4_^+^	342.23	342.26
**[M-H_2_O+Na]^+^**	C_17_H_32_N_4_O_3_Na^+^	363.23	363.30
**[M+H-2H_2_O]^+^**	C_17_H_31_N_4_O_2_^+^	323.24	323.24

## Supplementary Materials

The following are available online at https://www.mdpi.com/article/10.3390/nano11123176/s1, Figure S1. DLS analysis of magnetite nanoparticles with PABA shell, Figure S2. The EDX spectrum for Fe_3_O_4_-PABA, Figure S3. The N_2_ adsorption/desorption isotherm for Fe_3_O_4_-PABA, Figure S4. Pore size distributions for Fe_3_O_4_-PABA, Figure S5. TGA analysis of the Fe_3_O_4_-PABA-SiO_2_ nanoparticles after the water washing step, Figure S6. TGA analysis of the MW-NPS-1 nanoparticles, Figure S7. TGA analysis of the MW-NPS-2 nanoparticles, Figure S8. The N_2_ adsorption/desorption isotherm for MW-NPS-1, Figure S9. Pore size distributions for MW-NPS-1, Figure S10. The N_2_ adsorption/desorption isotherm for MW-NPS-2, Figure S11. Pore size distributions for MW-NPS-2, Figure S12. The FT-IR spectrum of MW-NPS-1, Figure S13. The EDX spectrum for MW-NPS-1, Figure S14. The FT-IR spectrum of MW-NPS-2, Figure S15. The EDX spectrum for MW-NPS-2, Figure S16. Magnetization curve of MW-NPS-2 nanoparticles, Figure S17. The FT-IR spectrum of MW-NPS-1-APS, Figure S18. The FT-IR spectrum of MW-NPS-2-APS, Figure S19. Magnetization curve of MW-NPS-2-APS, Figure S20. HR-MS analysis of peptide Val-Ile-Lys after cleavage from the NW-NPS-2-APS-HMBA nanostructured system, Figure S21. HR-MS analysis of peptide Val-Ile-Lys. Highlighting of ([M+H]^+^ + 1), ([M+H]^+^ + 2), ([M+H]^+^ + 3) type isotopic peaks derived from the protonated molecular ion [M+H]^+^, Figure S22. Identification in the MS-MS spectrum (from 359.34 monoisotopic peak precursor) of c_1_, y_1,_ z_1_ type fragments with *m/z* 117.43, 130.16, respectively 147.99, Figure S23. Identification in the MS-MS spectrum (from 359.34 monoisotopic peak precursor) of x_1_ and a_2_ type fragments with *m/z* 173.00, respectively 185.92, Figure S24. Identification in the MS-MS spectrum (from 359.34 monoisotopic peak precursor) of b_2_, c_2_ and z_2_ type fragments with *m/z* 213.90, 230.99, respectively 243.80, Figure S25. Identification in the MS-MS spectrum (from 359.34 monoisotopic peak precursor) of x_2_ and y_2_ type fragments with *m/z* 286.82 and 260.88, Figure S26. Identification in the MS-MS spectrum (from 359.34 monoisotopic peak precursor) of [M+H-H_2_O]^+^, [M+H-CO-H_2_O]^+^, [M+H-NH_3_]^+^, [M-H_2_O+Na]^+^, [M+H-2H_2_O]^+^ type fragments with *m/z* 341.26, 313.22, 342.26, 363.30, respectively 323.24
