# Supplementary material for: Microwave-Assisted Sol–Gel Preparation of the Nanostructured Magnetic System for Solid-Phase Synthesis
Source: Nanomaterials (Basel). 2021 Nov 24;11(12):3176. doi: 10.3390/nano11123176 (PMC8709383; doi:10.3390/nano11123176)
Supplement: Supplementary file 1 [file nanomaterials-11-03176-s001.zip › nanomaterials-1452922-supplementary.pdf]

Supplementary Materials

Microwave-Assisted Sol–Gel Preparation of the Nanostructured Magnetic System for Solid-Phase Synthesis

Daniela Istrati [1], Alina Moroșan [1], Raluca Stan [1,*], Bogdan Ștefan Vasile [2], Gabriel Vasilievici [3], Ovidiu Oprea [4], Georgiana Dolete [2], Bogdan Purcăreanu [5] and Dan Eduard Mihaiescu [1,*]

[1] Department of Organic Chemistry "Costin Nenitescu", Faculty of Applied Chemistry and Materials Science, Politehnica University of Bucharest, Bucharest 011061, Romania; daniela.istrati@upb.ro (D.I.); alina.morosan@upb.ro (A.M.)
[2] Department of Science and Engineering of Oxide Materials and Nanomaterials, Faculty of Applied Chemistry and Materials Science, Politehnica University of Bucharest, Bucharest 011061, Romania; vasile_bogdan_stefan@yahoo.com (B.Ș.V.); dolete.georgiana@gmail.com (G.D.)
[3] INCDCP-ICECHIM, 202 Spl. Independentei, Bucharest 011061, Romania; gabi.vasilievici@gmail.com
[4] Department of Inorganic Chemistry, Physical Chemistry and Electrochemistry, Faculty of Applied Chemistry and Materials Science, Politehnica University of Bucharest, Bucharest 011061, Romania; ovidiu73@yahoo.com
[5] S.C. BIOTEHNOS S.A., Gorunului Rue, No. 3–5, Ilfov 075100, Romania; bogdanpb89@gmail.com
* Correspondence: raluca.stan@upb.ro (R.S.) and dan.mihaiescu@upb.ro (D.E.M.); Tel.: +40-7400-95101 (R.S.); +40-7290-35718 (D.E.M.)

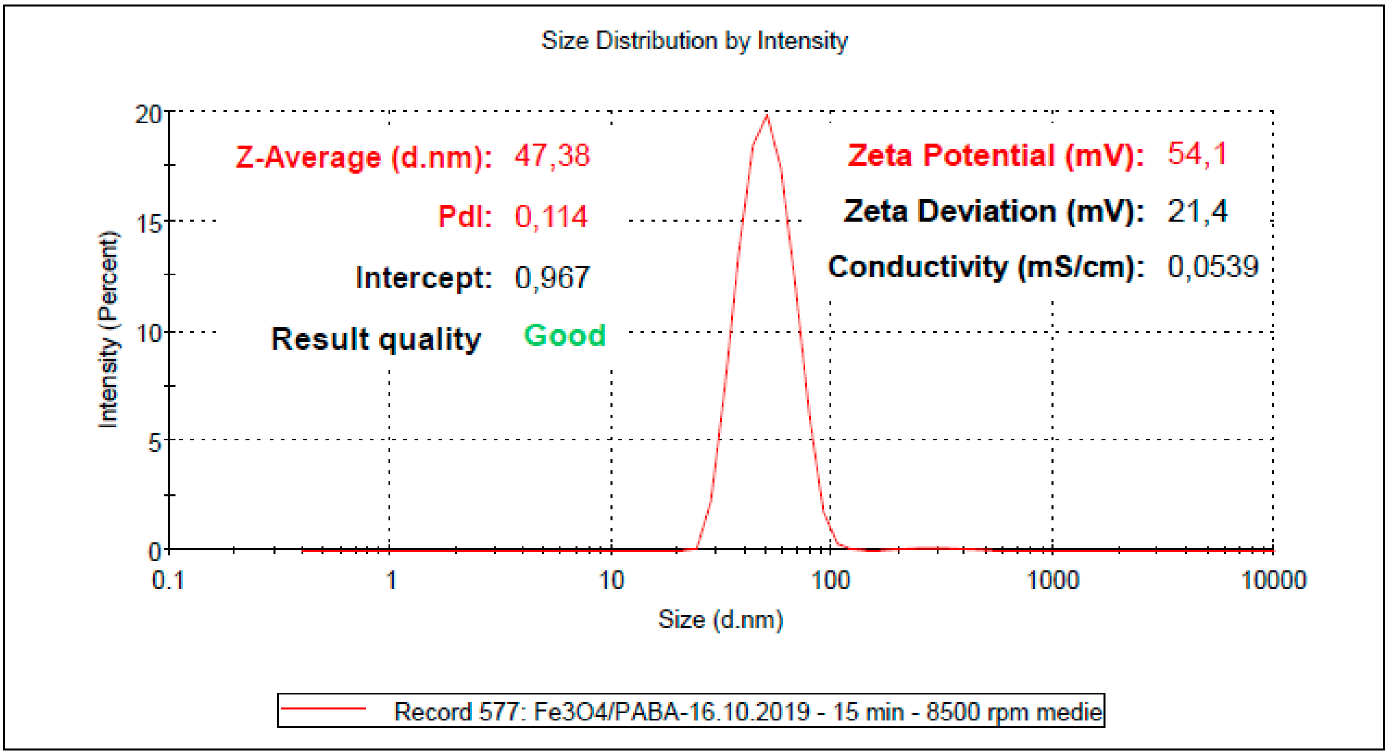

Figure S1. DLS analysis of magnetite nanoparticles with PABA shell.

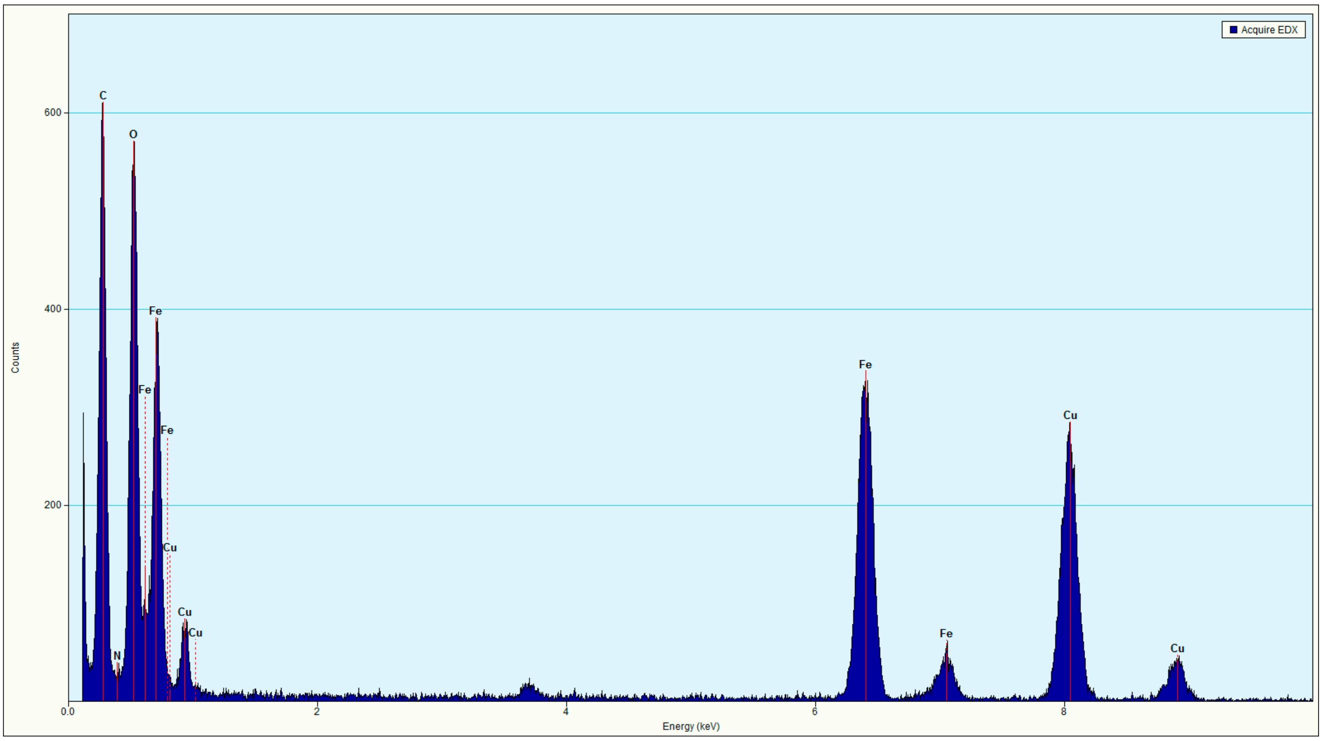

Figure S2. The EDX spectrum for Fe_3O_4-PABA.

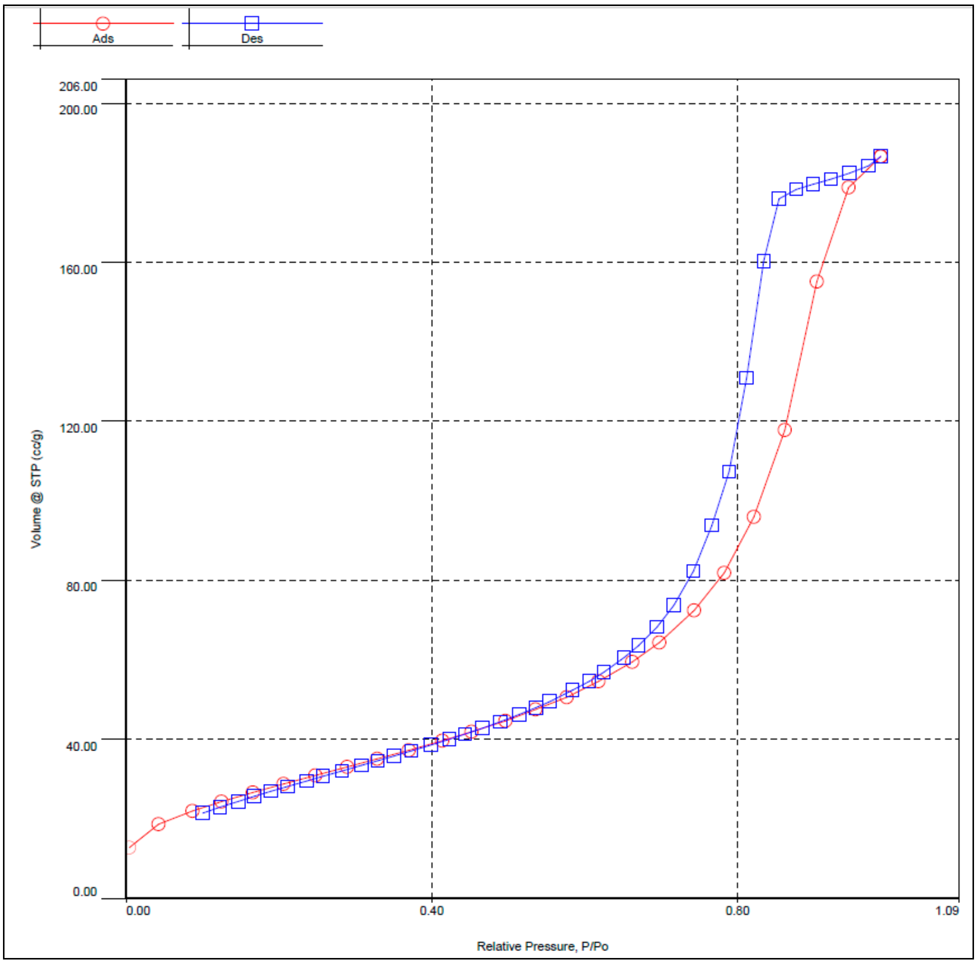

Figure S3. The N_2 adsorption/desorption isotherm for Fe_3O_4-PABA.

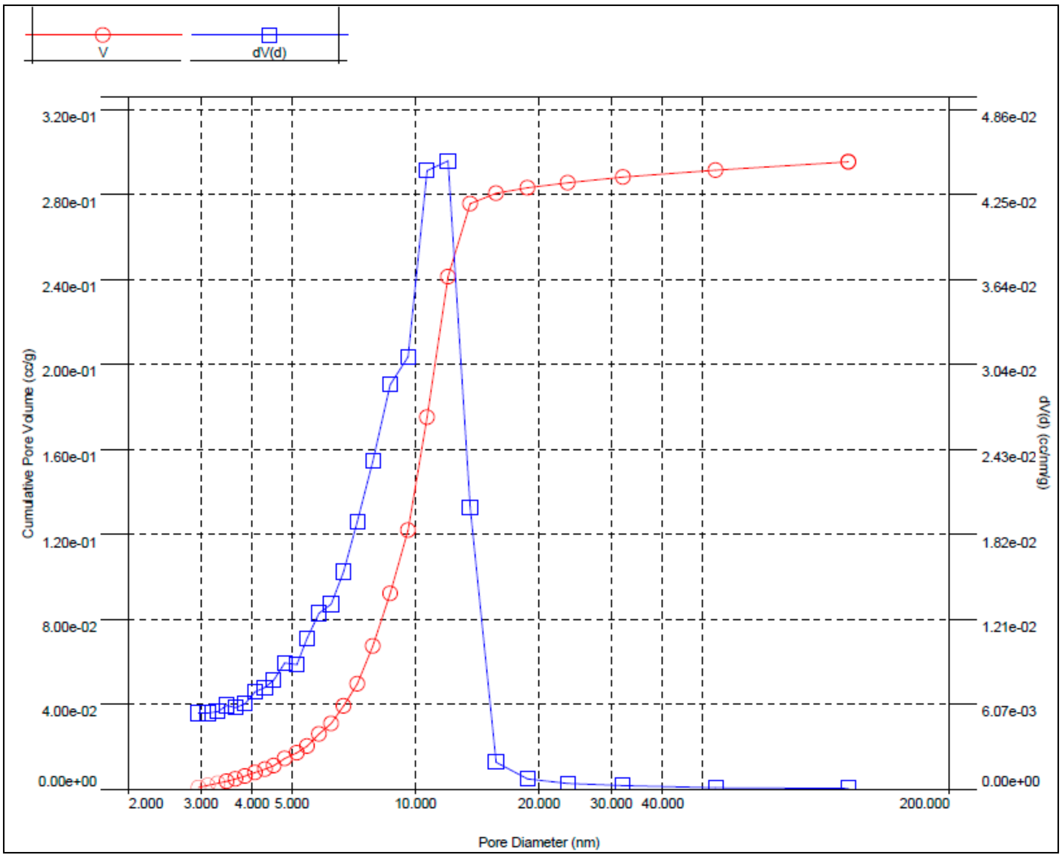

Figure S4. Pore size distributions for Fe_3O_4-PABA.

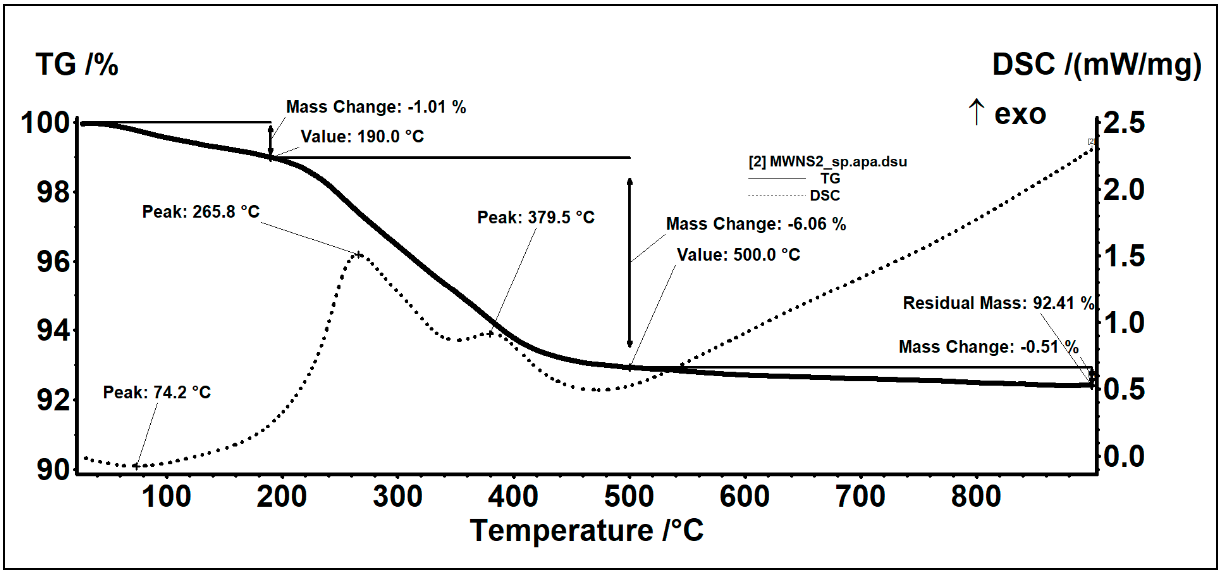

Figure S5. TGA analysis of the Fe_3O_4-PABA-SiO_2 nanoparticles after the water washing step.

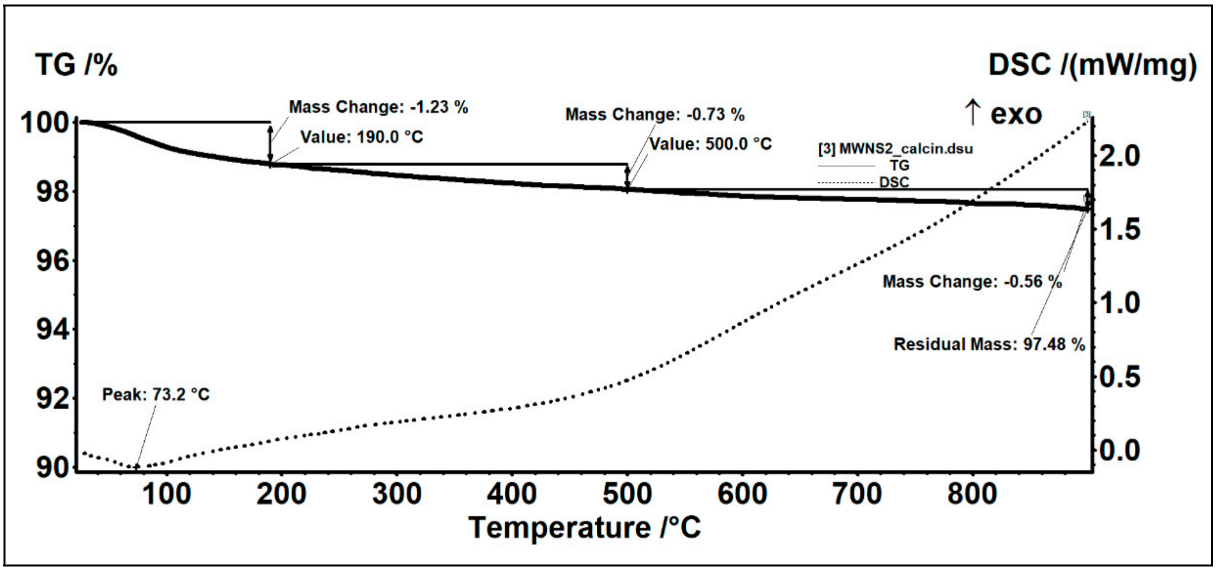

Figure S6. TGA analysis of the MW-NPS-1 nanoparticles.

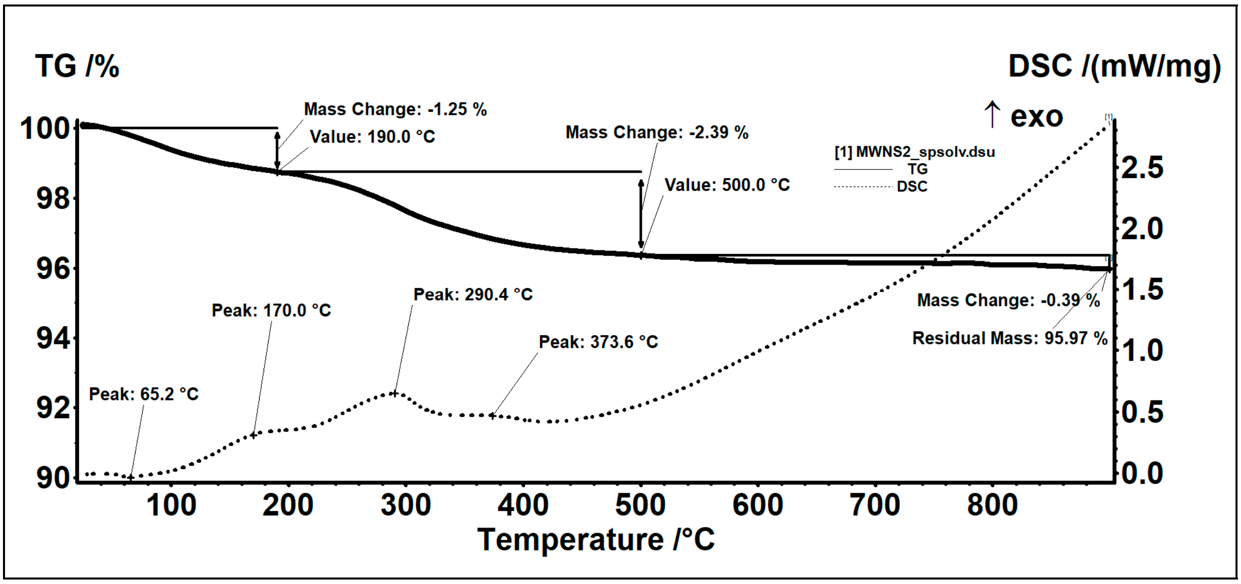

Figure S7. TGA analysis of the MW-NPS-2 nanoparticles.

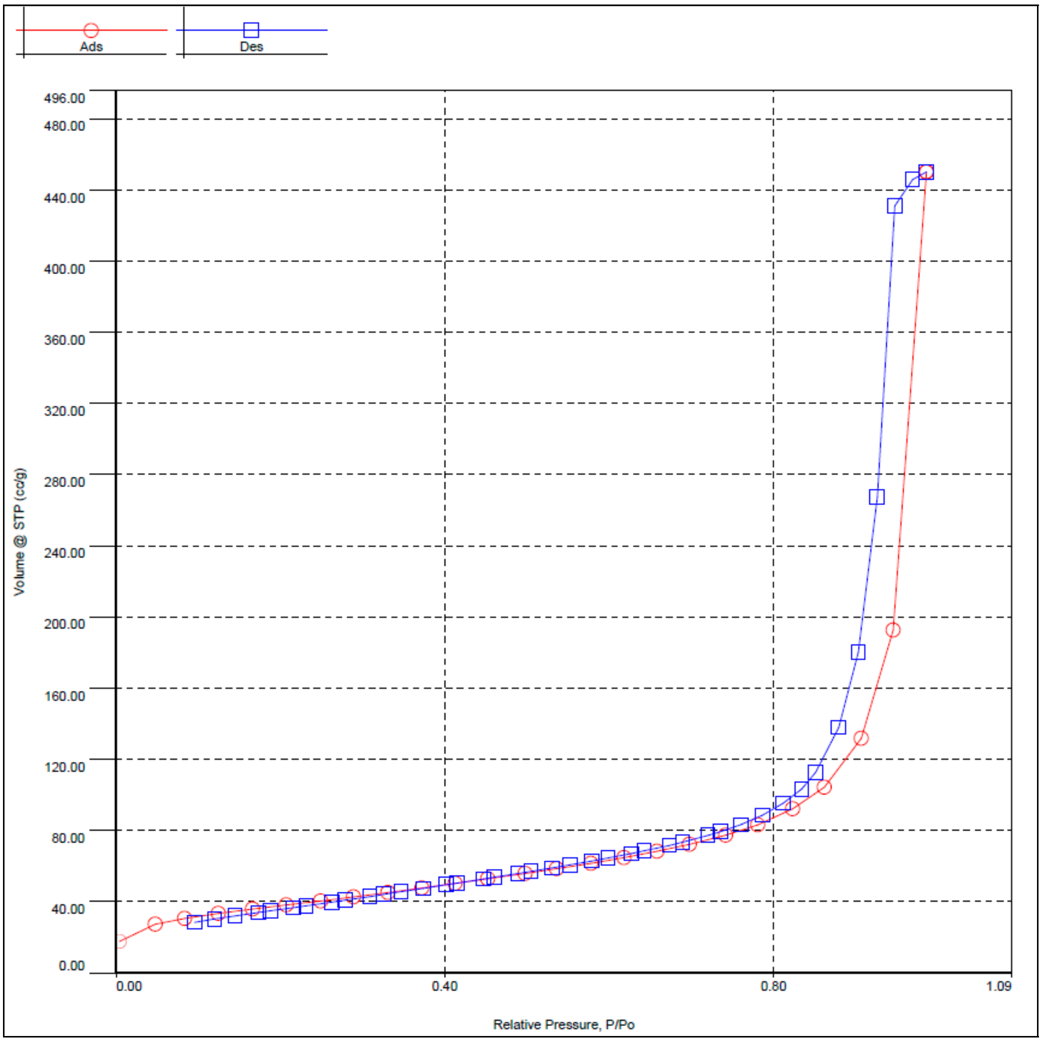

Figure S8. The N_2 adsorption/desorption isotherm for MW-NPS-1.

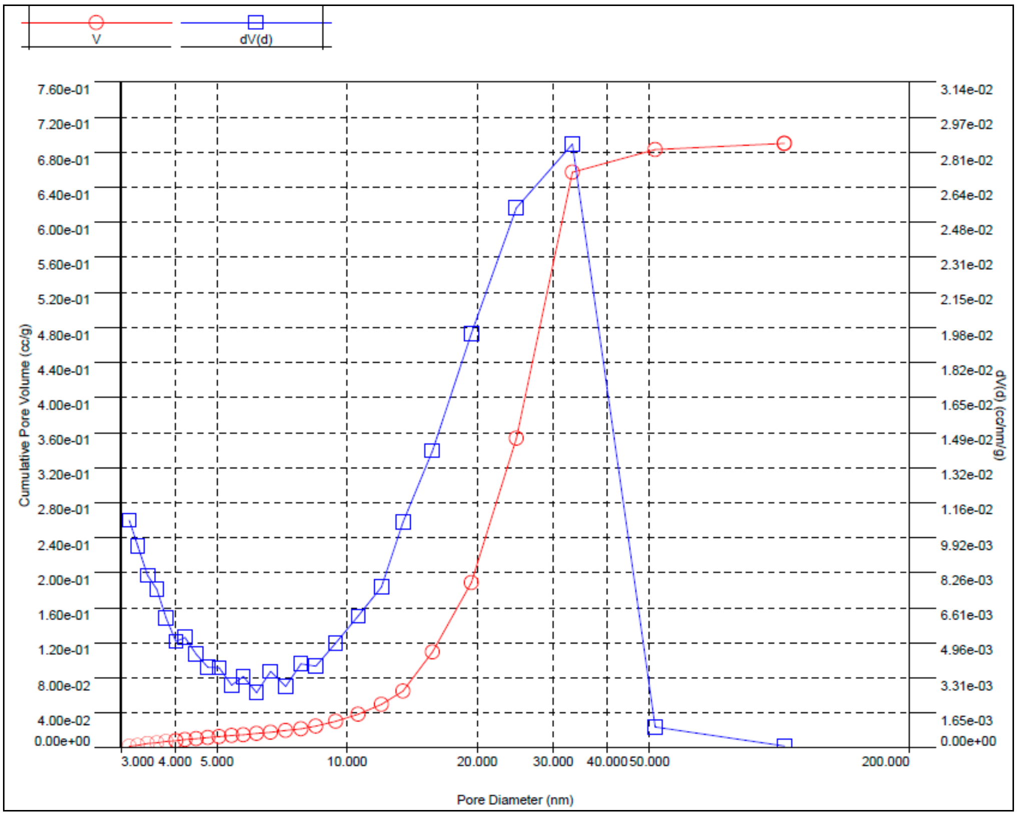

Figure S9. Pore size distributions for MW-NPS-1.

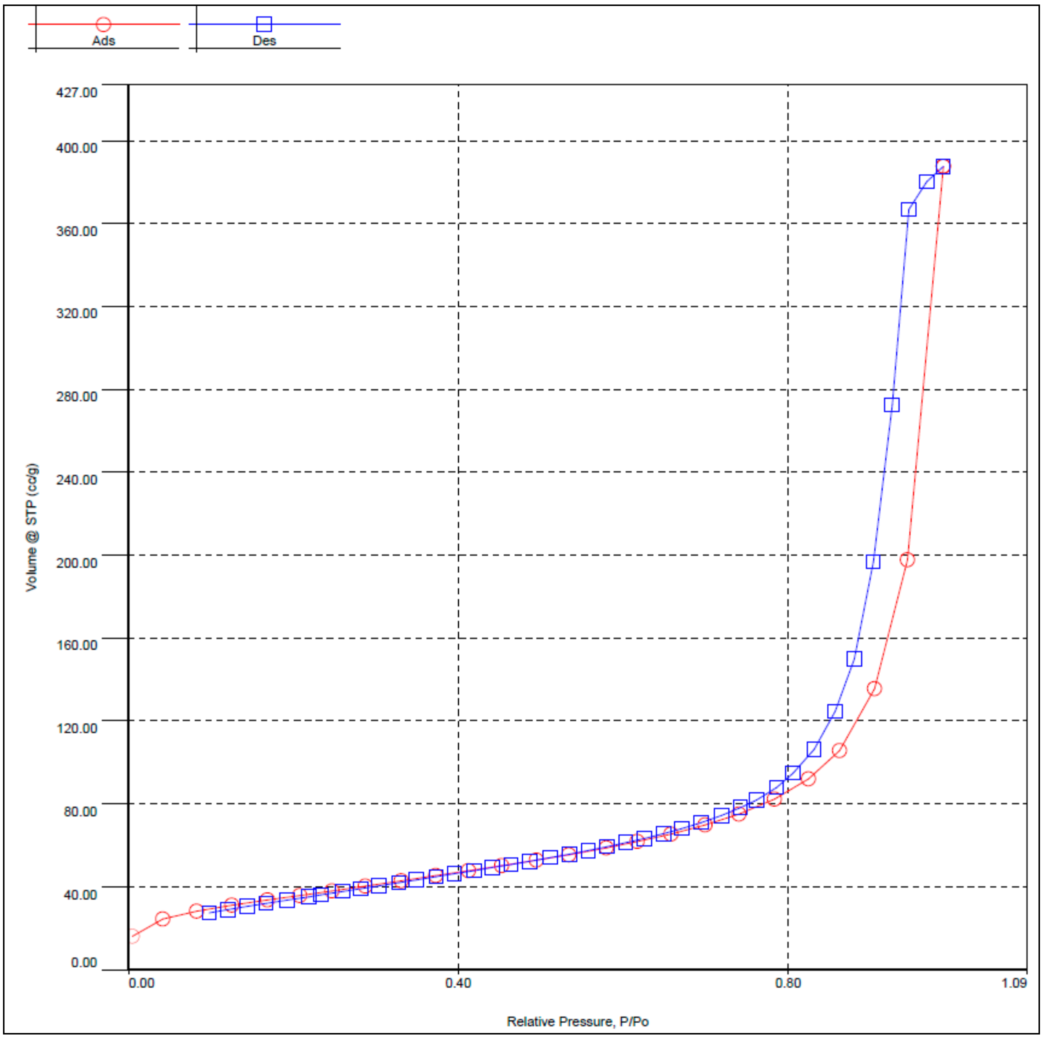

Figure S10. The N_2 adsorption/desorption isotherm for MW-NPS-2.

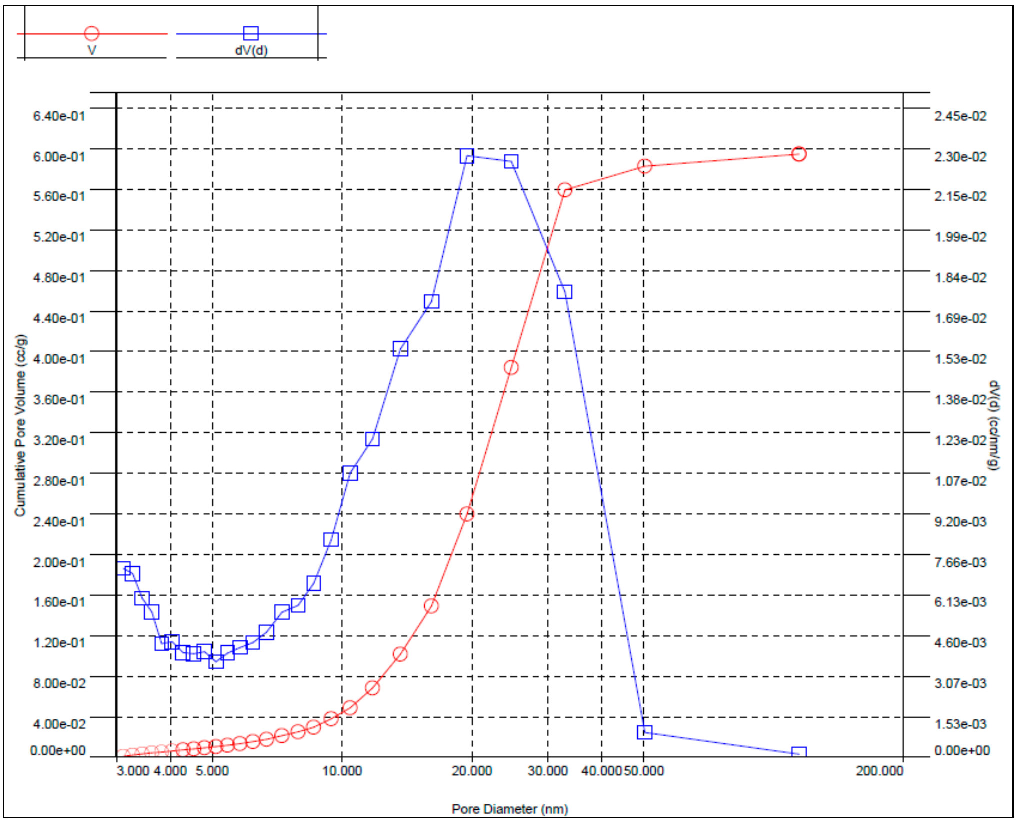

Figure S11. Pore size distributions for MW-NPS-2.

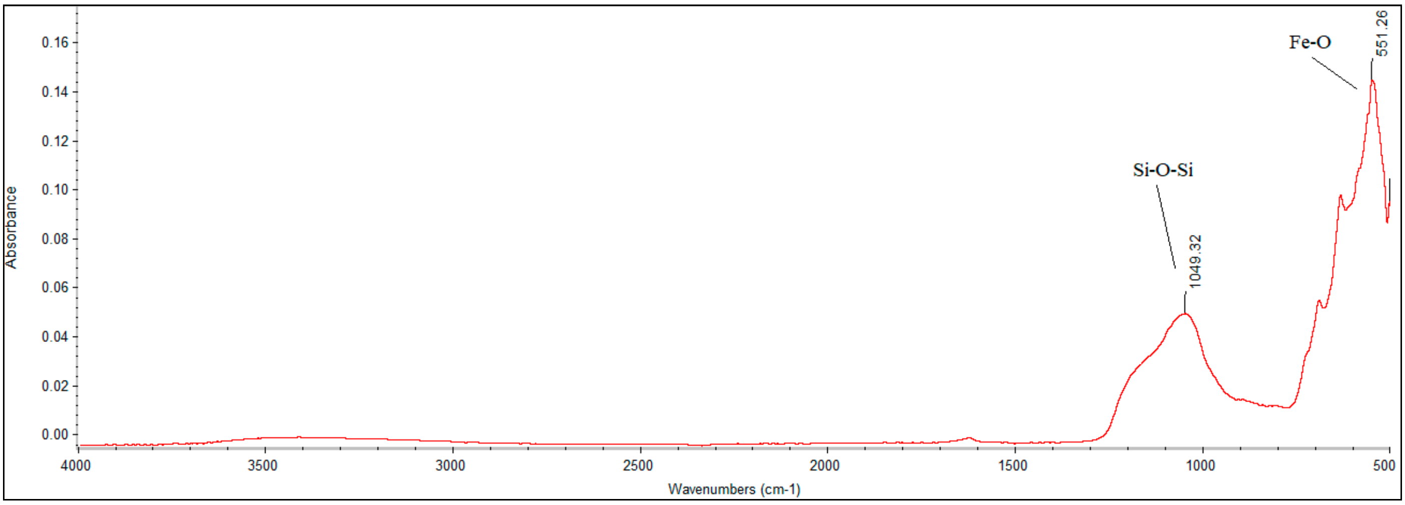

Figure S12. The FT-IR spectrum of MW-NPS-1.

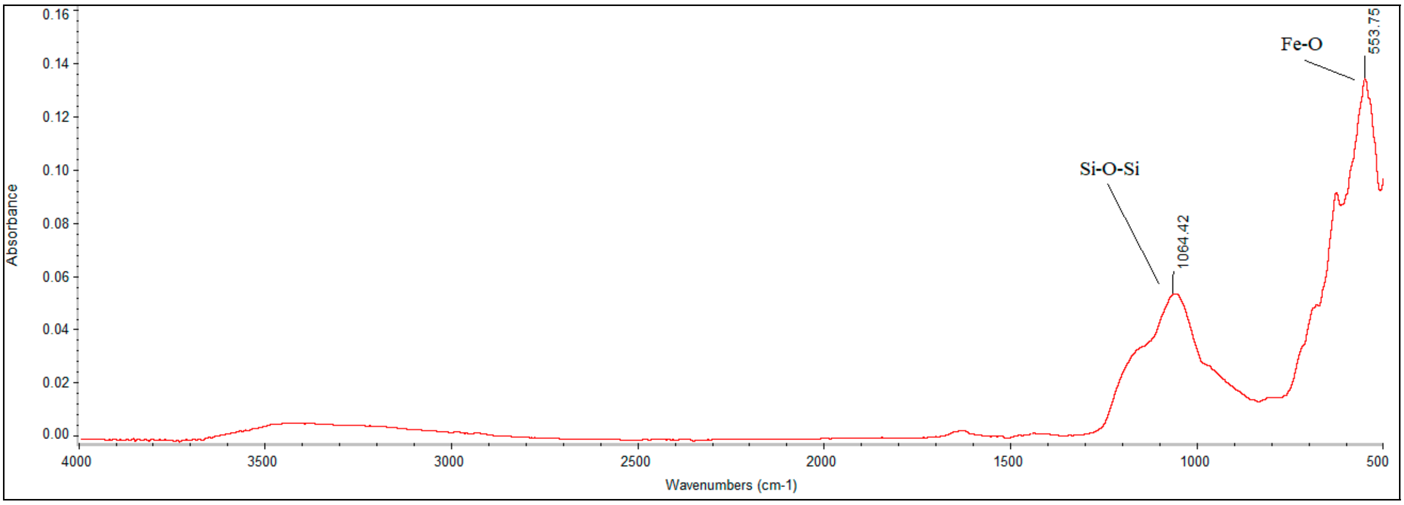

Figure S13. The FT-IR spectrum of MW-NPS-2.

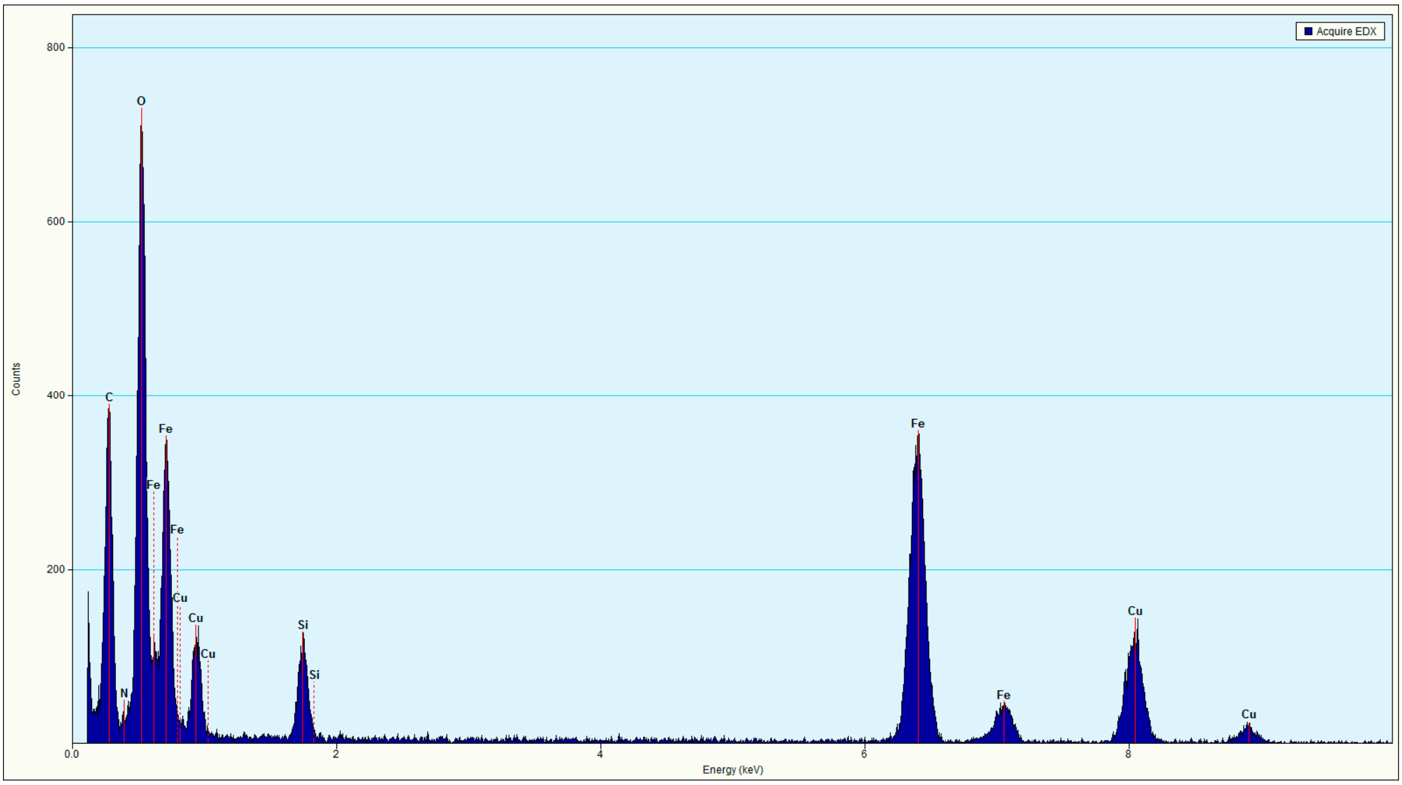

Figure S14. The EDX spectrum for MW-NPS-1.

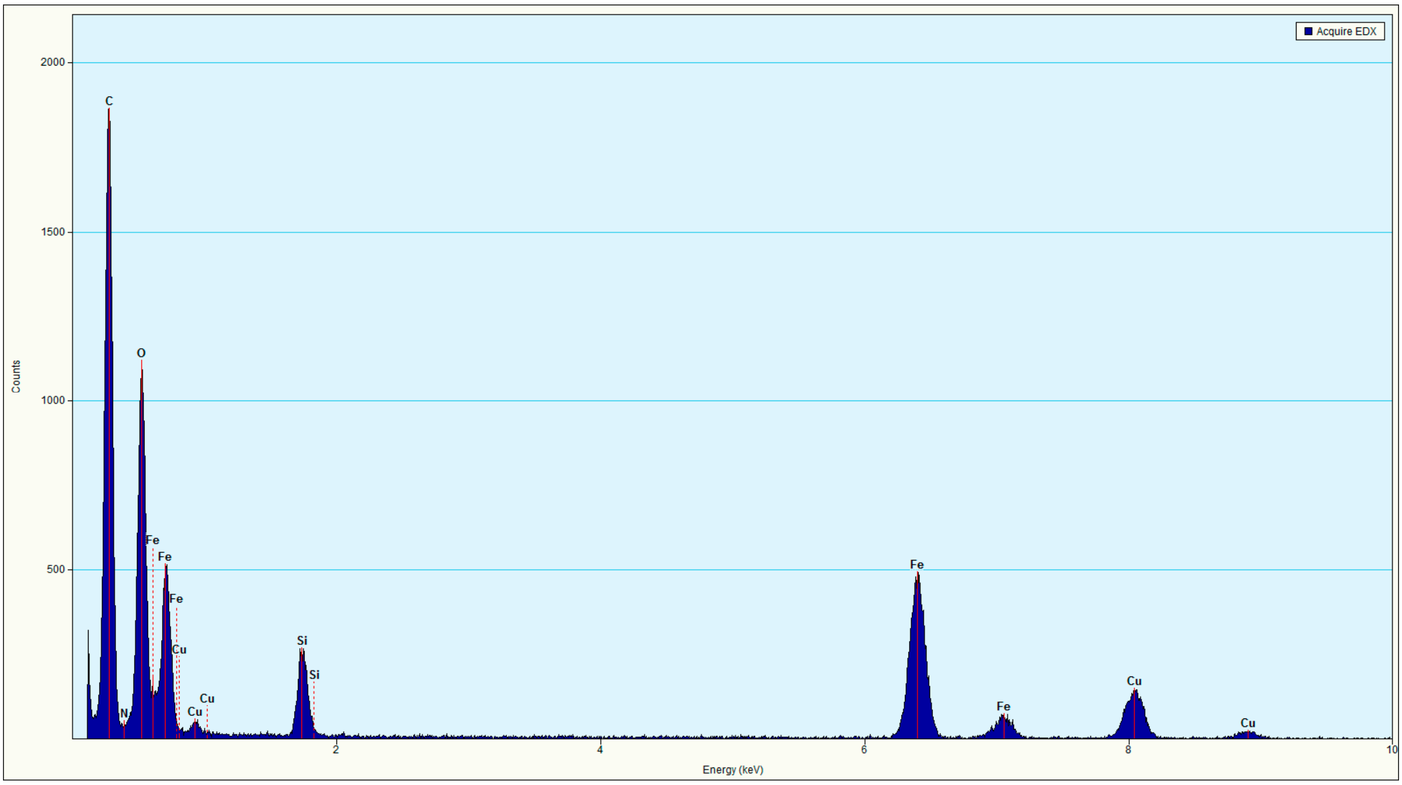

Figure S15. The EDX spectrum for MW-NPS-2.

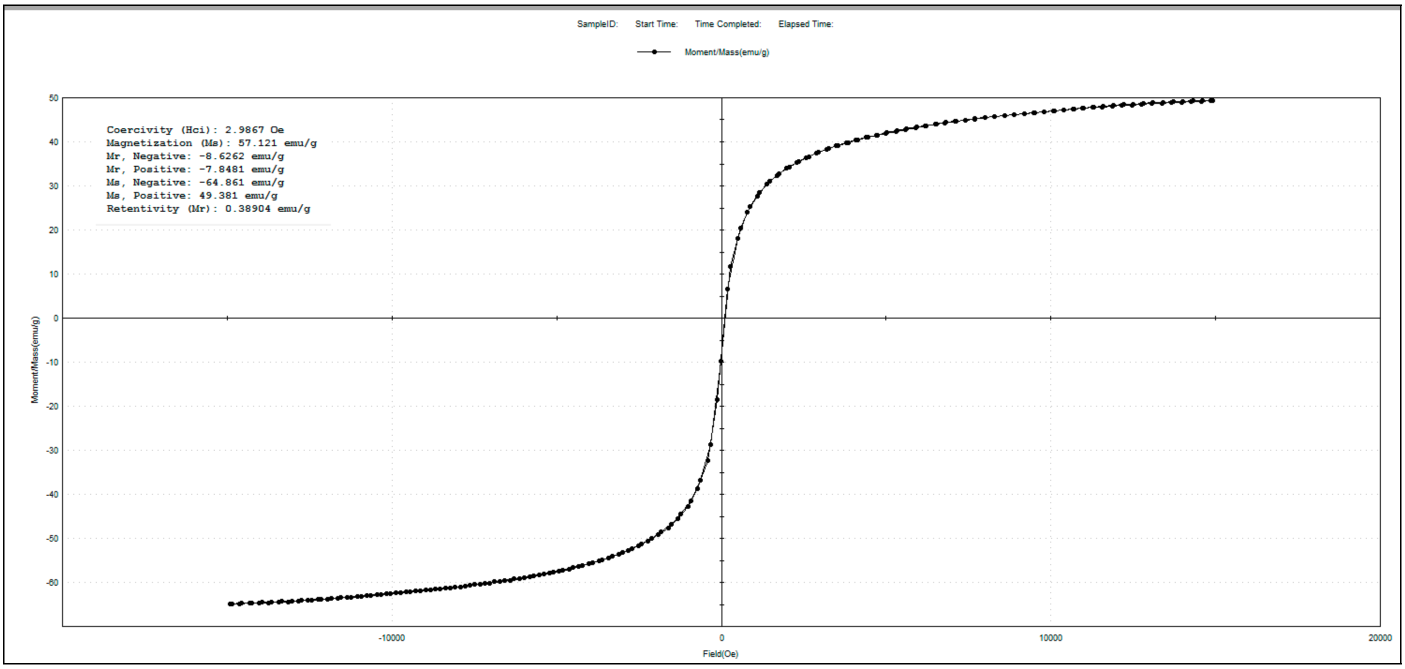

Figure S16. Magnetization curve of MW-NPS-2 nanoparticles.

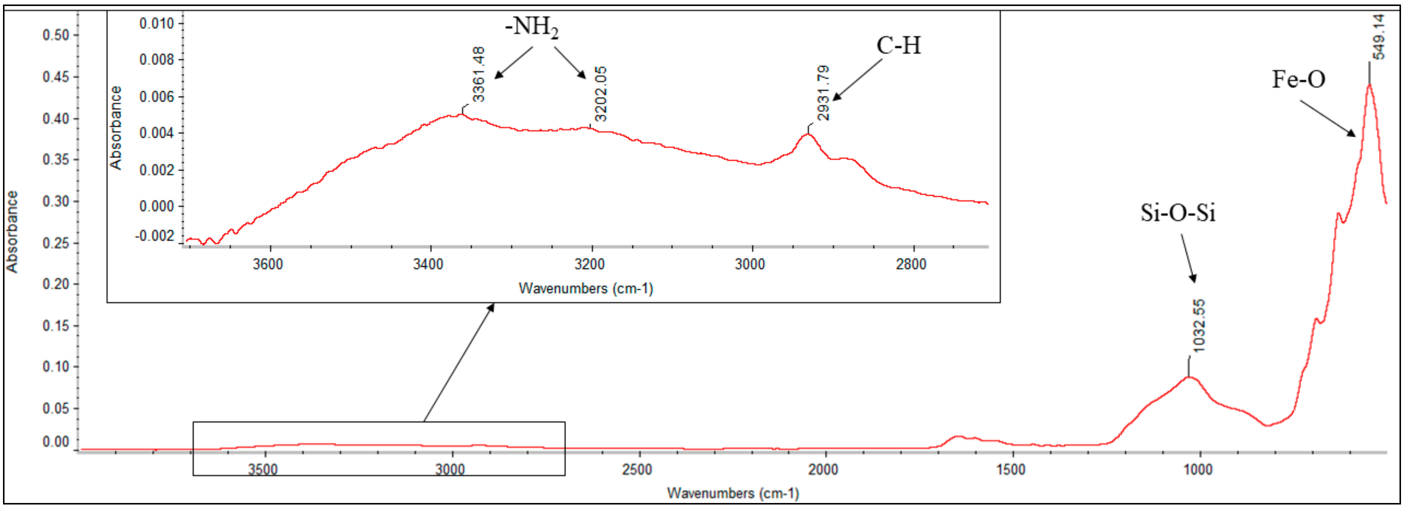

Figure S17. The FT-IR spectrum of MW-NPS-1-APS.

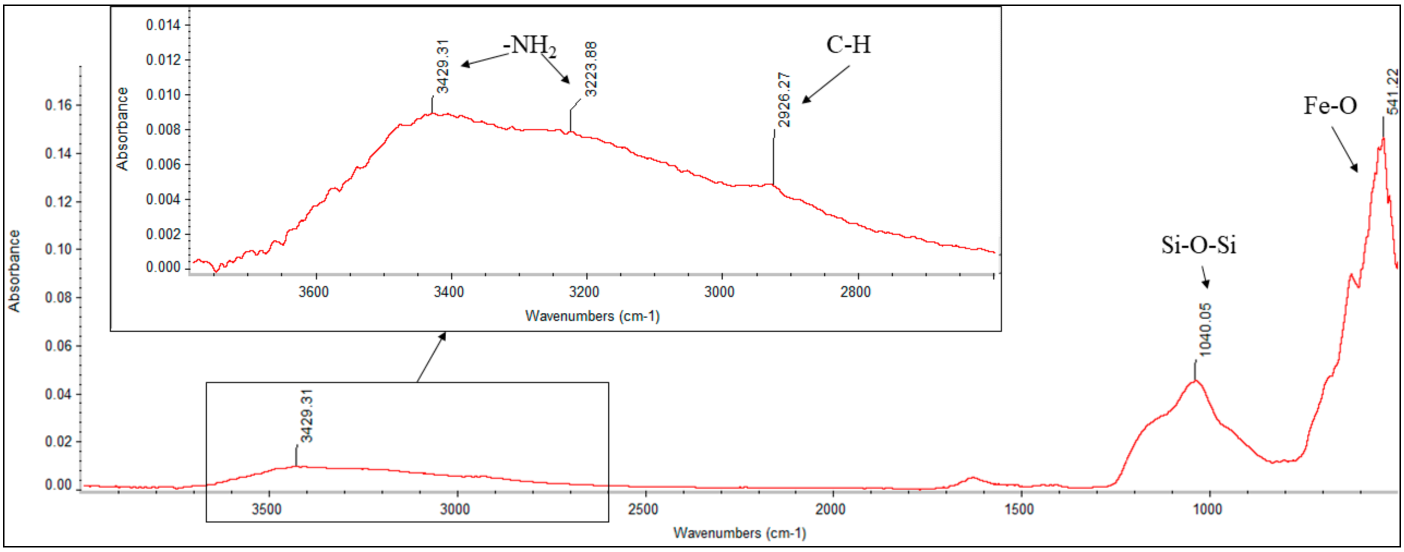

Figure S18. The FT-IR spectrum of MW-NPS-2-APS.

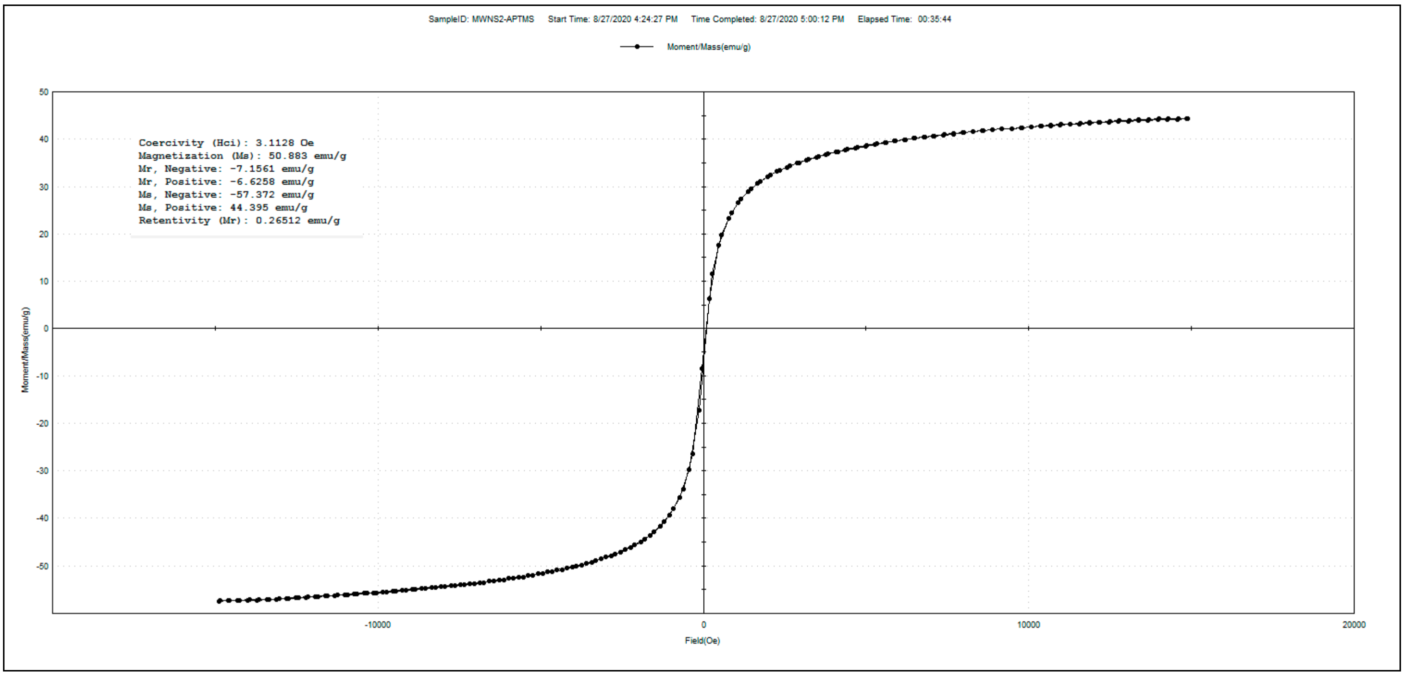

Figure S19. Magnetization curve of MW-NPS-2-APS.

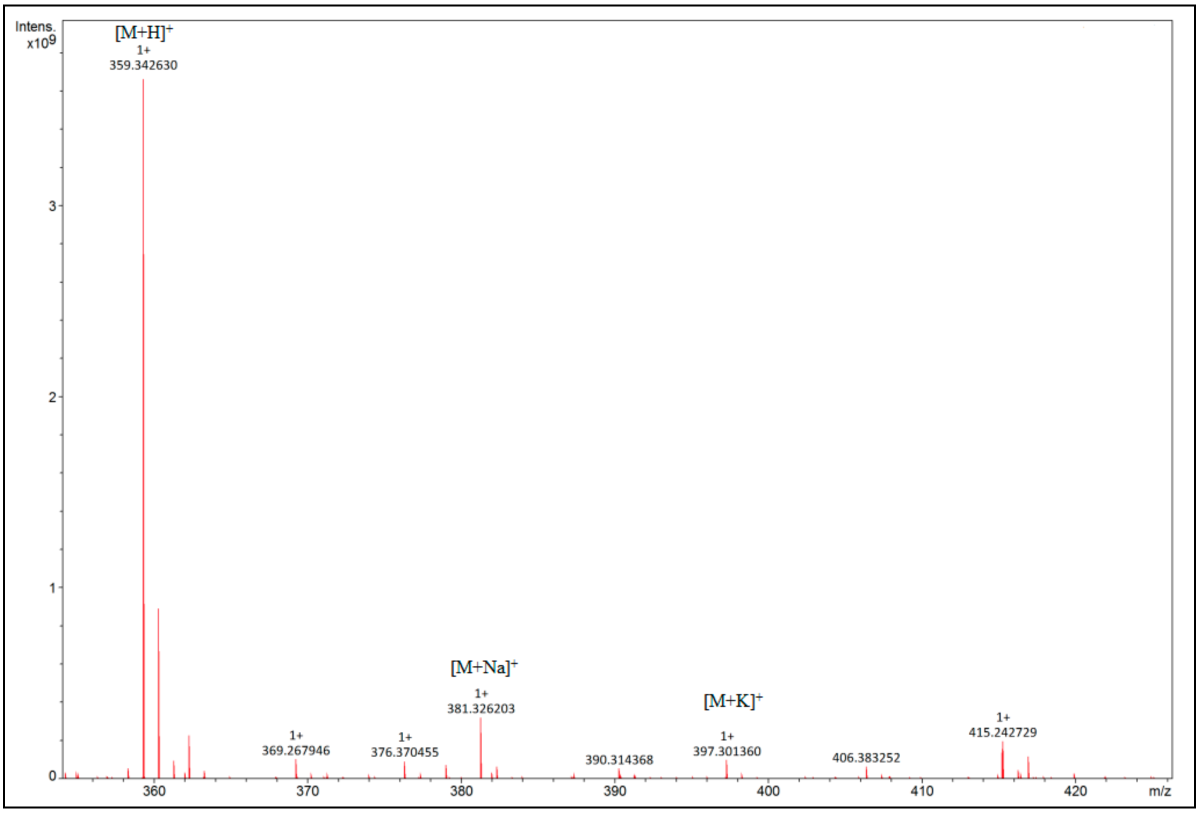

Figure S20. HR-MS analysis of peptide Val-Ile-Lys after cleavage from the NW-NPS-2-APS-HMBA nanostructured system.

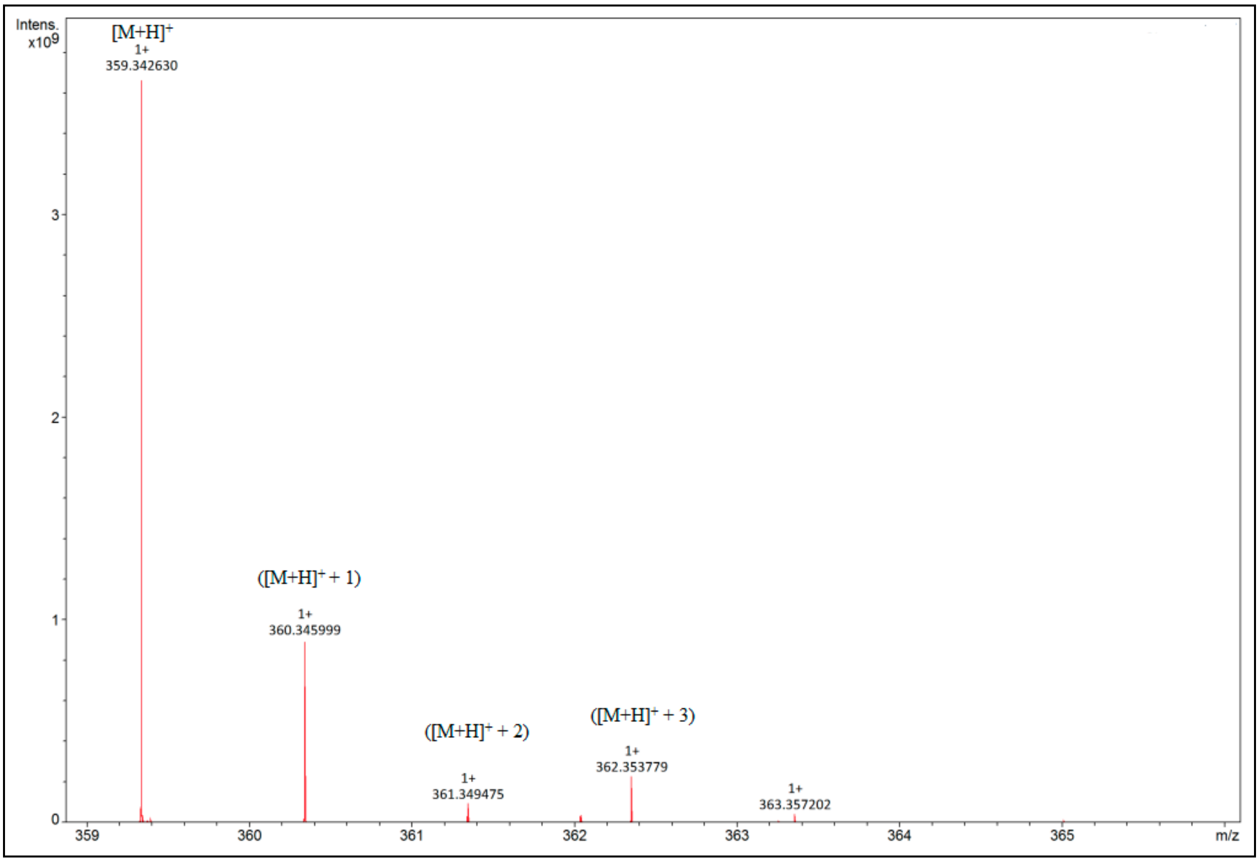

Figure S21. HR-MS analysis of peptide Val-Ile-Lys. Highlighting of ($[M+H]^+ + 1$), ($[M+H]^+ + 2$), ($[M+H]^+ + 3$) type isotopic peaks derived from the protonated molecular ion $[M+H]^+$.

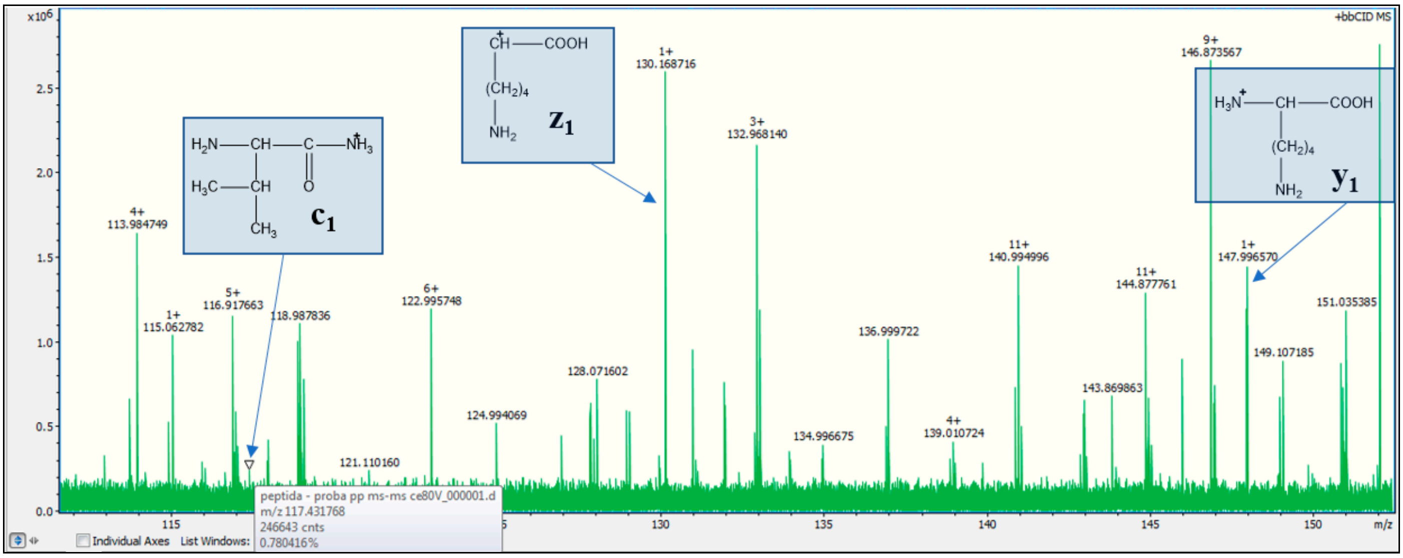

Figure S22. Identification in the MS-MS spectrum (from 359.34 monoisotopic peak precursor) of c_1, y_1, z_1 type fragments with m/z 117.43, 130.16, respectively 147.99.

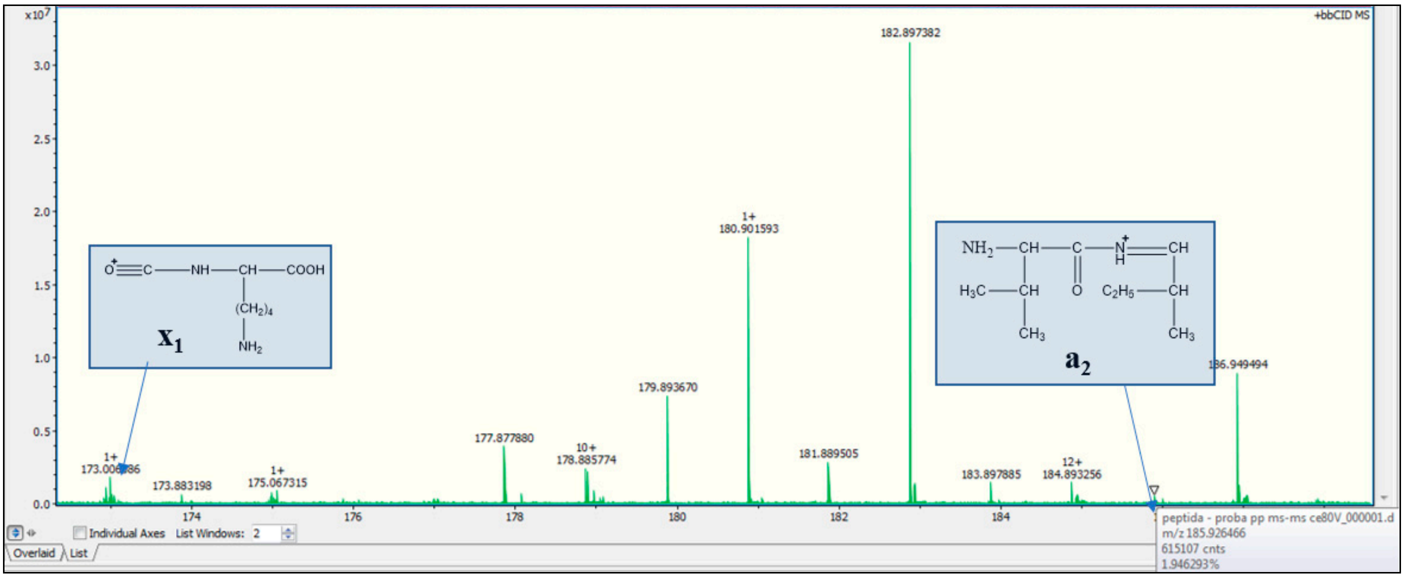

Figure S23. Identification in the MS-MS spectrum (from 359.34 monoisotopic peak precursor) of x_1 and a_2 type fragments with m/z 173.00, respectively 185.92.

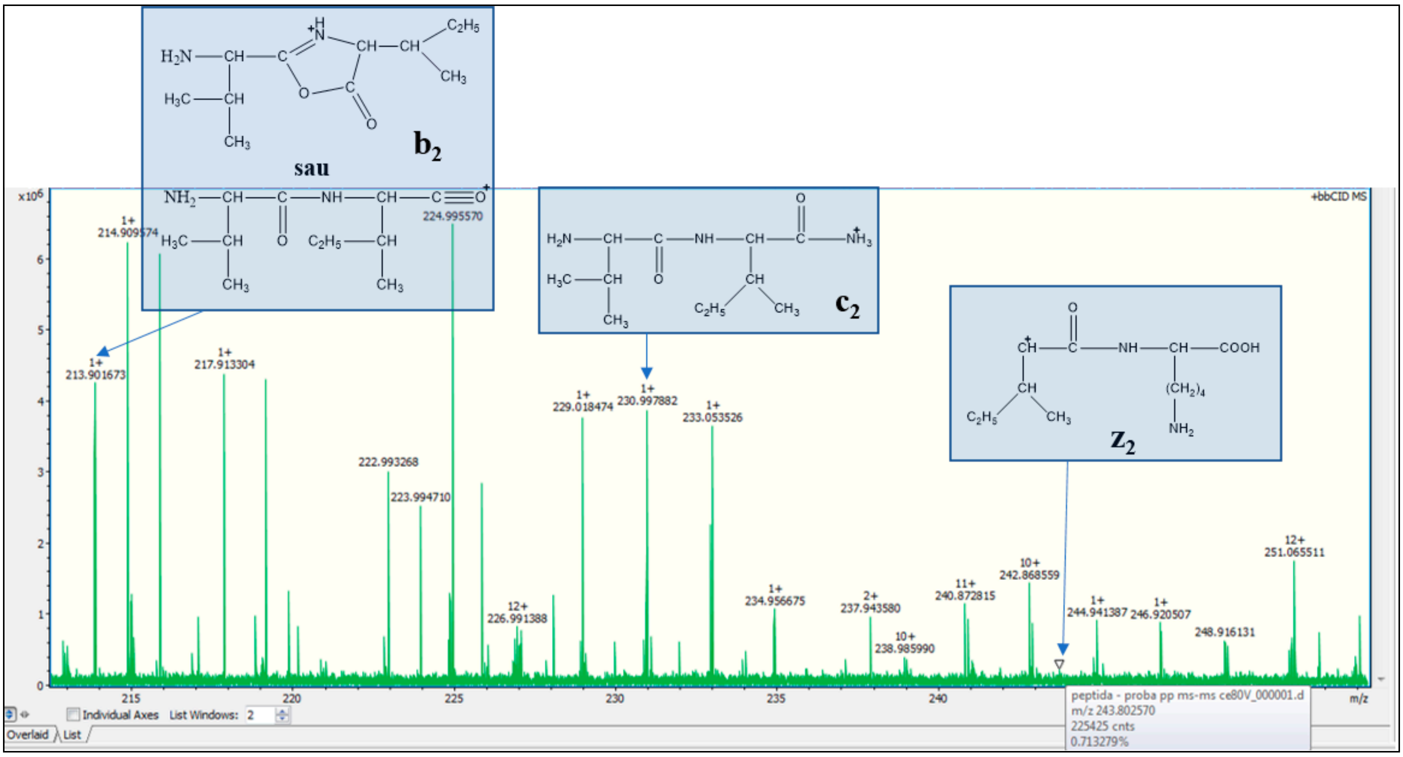

Figure S24. Identification in the MS-MS spectrum (from 359.34 monoisotopic peak precursor) of b_2, c_2 and z_2 type fragments with m/z 213.90, 230.99, respectively 243.80.

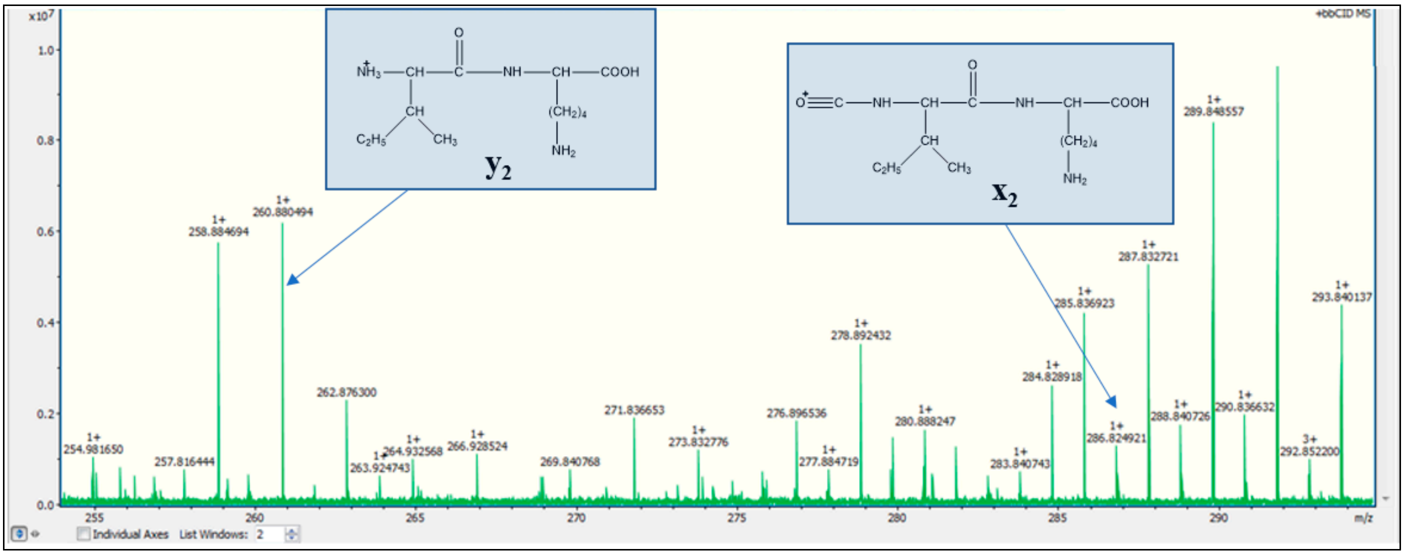

Figure S25. Identification in the MS-MS spectrum (from 359.34 monoisotopic peak precursor) of x_2 and y_2 type fragments with m/z 286.82 and 260.88.

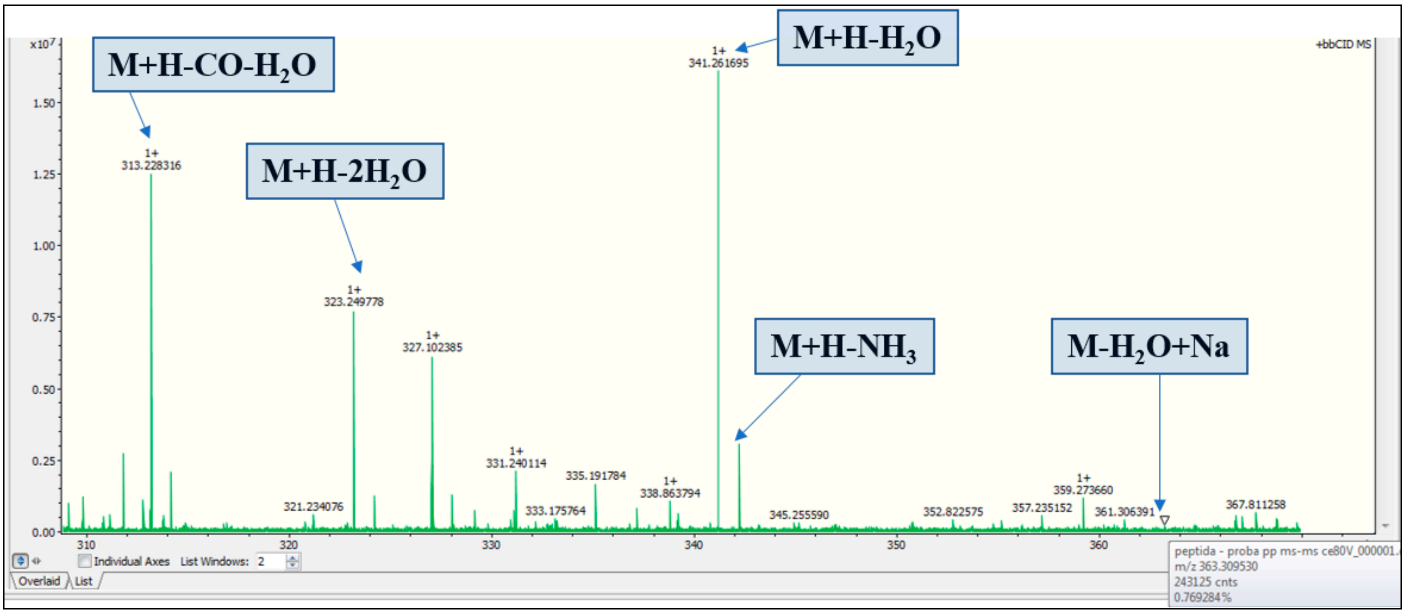

Figure S26. Identification in the MS-MS spectrum (from 359.34 monoisotopic peak precursor) of $[M+H-H_2O]^+$, $[M+H-CO-H_2O]^+$, $[M+H-NH_3]^+$, $[M-H_2O+Na]^+$, $[M+H-2H_2O]^+$ type fragments with m/z 341.26, 313.22, 342.26, 363.30, respectively 323.24.
